# ESCPE-1 mediates retrograde endosomal sorting of the SARS-CoV-2 host factor Neuropilin-1

**DOI:** 10.1073/pnas.2201980119

**Published:** 2022-06-13

**Authors:** Boris Simonetti, James L. Daly, Lorena Simón-Gracia, Katja Klein, Saroja Weeratunga, Carlos Antón-Plágaro, Allan Tobi, Lorna Hodgson, Philip A. Lewis, Kate J. Heesom, Deborah K. Shoemark, Andrew D. Davidson, Brett M. Collins, Tambet Teesalu, Yohei Yamauchi, Peter J. Cullen

**Affiliations:** ^a^School of Biochemistry, Faculty of Life Sciences, University of Bristol, Bristol BS8 1TD, United Kingdom;; ^b^Laboratory of Precision and Nanomedicine, Institute of Biomedicine and Translational Medicine, University of Tartu, Tartu 50411, Estonia;; ^c^School of Cellular and Molecular Medicine, Faculty of Life Sciences, University of Bristol, Bristol BS8 1TD, United Kingdom;; ^d^Institute for Molecular Bioscience, The University of Queensland, St. Lucia, QLD 4072, Australia;; ^e^Proteomics Facility, School of Biochemistry, Faculty of Life Sciences, University of Bristol, Bristol BS8 1TD, United Kingdom;; ^f^School of Biochemistry and BrisSynBio Centre, Faculty of Life Sciences, University of Bristol, Bristol BS8 1TD, United Kingdom

**Keywords:** endosome, Neuropilin-1, SARS-CoV-2, COVID-19, sorting nexin

## Abstract

Endosomal sorting maintains cellular homeostasis by recycling transmembrane proteins and associated proteins and lipids (termed “cargoes”) from the endosomal network to multiple subcellular destinations, including retrograde traffic to the *trans*-Golgi network (TGN). Viral and bacterial pathogens subvert retrograde trafficking machinery to facilitate infectivity. Here, we develop a proteomic screen to identify retrograde cargo proteins of the endosomal SNX-BAR sorting complex promoting exit 1 (ESCPE-1). Using this methodology, we identify Neuropilin-1 (NRP1), a recently characterized host factor for severe acute respiratory syndrome coronavirus 2 (SARS-CoV-2) infection, as a cargo directly bound and trafficked by ESCPE-1. ESCPE-1 mediates retrograde trafficking of engineered nanoparticles functionalized with the NRP1-interacting peptide of the SARS-CoV-2 spike (S) protein. CRISPR-Cas9 deletion of ESCPE-1 subunits reduces SARS-CoV-2 infection levels in cell culture. ESCPE-1 sorting of NRP1 may therefore play a role in the intracellular membrane trafficking of NRP1-interacting viruses such as SARS-CoV-2.

## Significance

To facilitate internalization into the host cell’s endosomal network, viruses recognize cell surface receptors and host factors. Upon network entry, the dynamic process of endosomal sorting underpins infection by regulating the intracellular trafficking and turnover of host factors and associated pathogenic proteins. Here, we identify Neuropilin-1, a host factor for infection by severe acute respiratory syndrome coronavirus 2 (SARS-CoV-2), Epstein-Barr virus (EBV), and human T cell lymphotropic virus type 1 (HTLV-1), as a direct cargo sorted by the endosomal SNX-BAR sorting complex promoting exit 1 (ESCPE-1). Disruption of this process perturbs NRP1-dependent endosomal trafficking of the SARS-CoV-2 spike protein, thus revealing an intracellular pathway that may contribute to the mechanism of SARS-CoV-2 infection and a number of other pathogenic viruses.

**Fig. 1.**
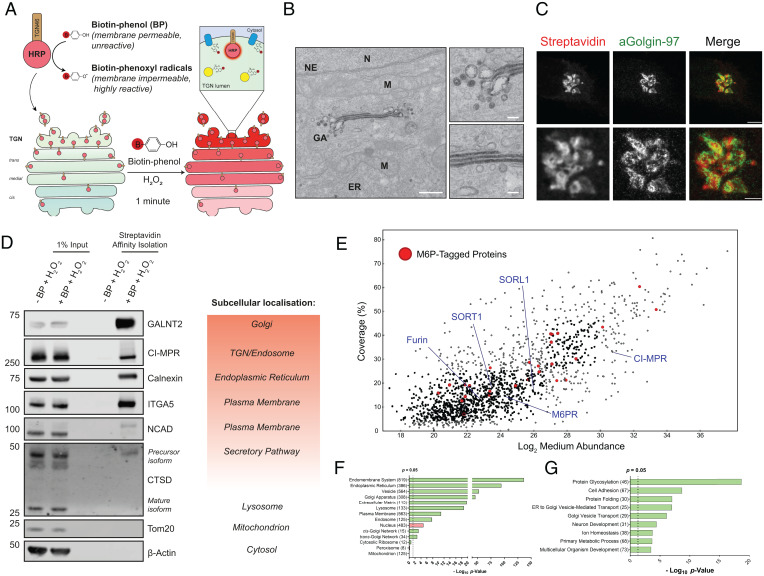
Development of a methodology to biotinylate TGN-resident proteins. (*A*) Schematic of HRP-TGN46 biotinylation labeling of endogenous TGN-resident proteins. In the presence of biotin-phenol and hydrogen peroxide, HRP catalyses the formation of membrane-impermeable biotin-phenoxyl that covalently label nearby endogenous proteins. (*B*) Transmission electron micrograph of HRP-TGN46–expressing HeLa cells incubated with DAB and H_2_O_2_ for 10 min. (Scale bar, 500 nm; zoom scale bar, 100 nm.) ER, endoplasmic reticulum; GA, Golgi apparatus; M, mitochondrion; N, nucleus; NE, nuclear envelope. (*C*) STED microscopy of HRP-TGN46–expressing HeLa cells following biotinylation, stained with fluorescent streptavidin and immunofluorescence labeling of Golgin-97. (Scale bars, 5 µm; *Insets*, 2 µm.) (*D*) Streptavidin affinity isolation of biotinylated proteins from total cell lysate of HRP-TGN46–expressing cells incubated with H_2_O_2_ in the presence or absence of BP, and their corresponding known subcellular localization. (*E*) Scatterplot of all HRP-TGN46–labeled proteins, with known mannose-6-phosphate–tagged proteins overlaid in red, and example TGN retrograde cargoes highlighted by blue labels; *n* = 5 independent repeats. (*F–G*) Graphical representation of overrepresentation of gene ontology cellular compartments (*F*) and biological processes (*G*) within the HRP-TGN46–proteome according to their calculated *P* value. Overrepresented gene ontology terms are colored green, and underrepresented terms are colored red. The numbers of identified proteins belonging to each category are provided within parentheses.

Retrograde transport from endosomes to the *trans*-Golgi network (TGN) diverts integral proteins away from lysosomal degradation to facilitate a variety of cellular functions (1) and is exploited by multiple pathogens (2–8). Despite technical advances into the study of this process (9–13), the broad array of cargoes that traverse this pathway and the mechanistic basis of their sorting largely remains elusive due to the lack of tools for the unbiased interrogation of the dynamic TGN proteome. To overcome this, we engineered a peroxidase-based proximity biotinylation methodology by fusing horseradish peroxidase (HRP) to the luminal terminus of TGN46, a representative single-pass type I transmembrane protein that predominantly localizes to the TGN (14). Following incubation with the membrane-permeable precursor biotin-phenol (BP) and a brief 60-s addition of hydrogen peroxide (H_2_O_2_), HRP generates short-lived, membrane-impermeable biotin-phenoxyl radicals that irreversibly biotinylate vicinal soluble proteins and the luminal domains of transmembrane proteins (Fig. 1*A*) (15).

In a HeLa cell line stably expressing HRP-TGN46 (*SI Appendix*, Fig. S1*A*), biotinylation was exclusively observed upon the presence of HRP-TGN46, BP, and H_2_O_2_ (*SI Appendix*, Fig. S1*B*). By either light microscopy, visualized by fluorescent streptavidin staining, or electron microscopy, visualized by oxidative polymerization of the electron-dense 3,3′-diaminobenzidine (DAB), we observed predominant labeling of the TGN upon activation of HRP-TGN46 (Fig. 1 *B* and *C* and *SI Appendix*, Fig. S1 *C* and *D*). The streptavidin-based affinity isolation of whole cell lysates following biotinylation showed specific labeling of endogenous proteins spanning the secretory pathway, including the Golgi-localized precursor isoform of the lysosomal hydrolase cathepsin D (CTSD); the glycosylation enzyme polypeptide *N*-acetylgalactosaminyltransferase 2 (GALNT2); the retrograde TGN-resident cargo cation-independent mannose 6-phosphate receptor (CI-MPR); the endoplasmic reticulum (ER) protein calnexin; and cell surface receptors such as integrin-α5 (ITGA5) and *N*-cadherin (NCAD), that traverse the biosynthetic pathway en route to the plasma membrane (Fig. 1*D*). Stable isotope labeling of amino acids in cell culture (SILAC)-based mass spectrometry revealed a list of 1,237 proteins reproducibly enriched by streptavidin affinity isolation following HRP-TGN46 labeling (Fig. 1*E* and *SI Appendix*, Fig. S1 *E* and *F* and Datasets S1 and S2). Gene ontology analysis demonstrated an enrichment of cellular components corresponding to the biosynthetic pathway and the interface of the TGN with the endolysosomal network, including a 74% coverage of TGN-resident mannose-6-phoshphate–tagged proteins (16), and validated retrograde transmembrane cargoes, including Furin, CI-MPR, M6PR, SORT1, and SORL1 (Fig. 1 *F* and *G* and Datasets S3–S5).

**Fig. 2.**
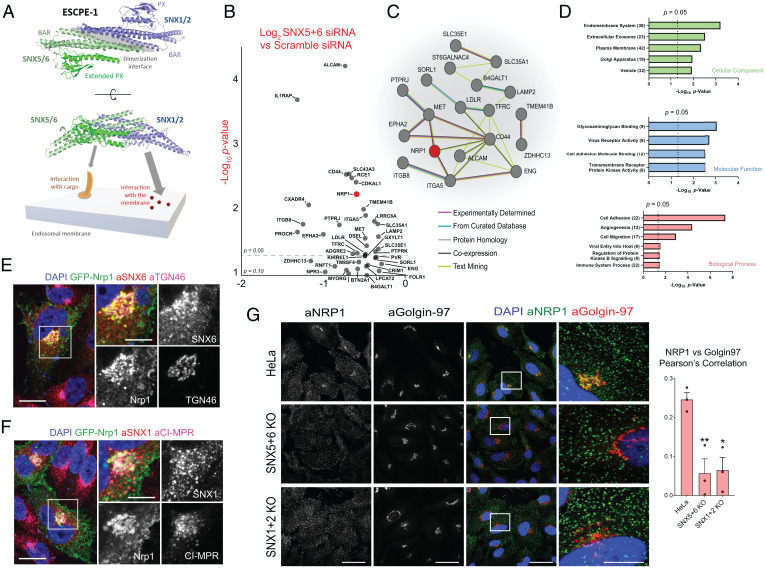
A proteomic screen for ESCPE-1 retrograde cargoes identifies NRP1. (*A*) Schematic of ESCPE-1 dimerization and coincidence detection of endosomal membranes and transmembrane cargo. (*B*) Volcano plot of transmembrane proteins depleted from the HRP-TGN46–labeled proteome upon SNX5 + SNX6 suppression by < Log_2_ −0.26, *P* value < 0.1 by SILAC proteomics. *P* = 0.05 is indicated by a dotted line. NRP1 is highlighted in red; *n* = 4 independent repeats. (*C*) STRING network analysis of the transmembrane proteins presented in *B*. The legend indicates the level of evidence for each protein–protein association. (*D*) Gene ontology analysis of the transmembrane proteins identified in *B*, classified by cellular component, molecular function, and biological process. Graphs represent the statistical significance of category enrichment, with a dotted line representing *P* = 0.05. The numbers of proteins in each category are provided within parentheses. (*E* and *F*) HeLa cells expressing GFP-Nrp1 were costained with anti-SNX6 and anti-TGN46 antibodies (*E*) or anti-SNX1 and anti-CI-MPR antibodies (*F*). (Scale bar, 20 µm; zoom, 10 µm.) (*G*) Immunofluorescence staining of endogenous NRP1 in HeLa WT cells, SNX5 + 6 KO HeLa cells and SNX1 + 2 KO HeLa cells. (Scale bar, 50 µm; *Insets*, 5 µm.) Pearson’s correlation quantification of the colocalization of NRP1 and Golgin-97 upon ESCPE-1 depletion. Ordinary one-way ANOVA with Dunnett’s multiple comparison’s tests; *n* = 3 independent repeats. WT vs. SNX5 + 6 KO *P* = 0.0084, WT vs. SNX1 + 2 KO *P* = 0.0103. *P < 0.05, **P < 0.01.

ESCPE-1 is an endosomal coat complex implicated in the retrograde trafficking of transmembrane proteins such as the CI-MPR (13, 17–19). ESCPE-1 consists of a heterodimer of sorting nexin 1 (SNX1) or SNX2, which are functionally redundant, associated with either SNX5 or SNX6, which are also functionally redundant, and mediate direct binding to the cytosolic tails of transmembrane cargo (17–20) (Fig. 2*A*). ESCPE-1 cargoes possess a ФxΩxФx_n_Ф sorting motif in their cytosolic tail (whereby Ф corresponds to hydrophobic residues and Ω represents a central aromatic residue) that folds into a β-hairpin structure (comprising two strands, denoted βA and βB, interspaced by a flexible loop of variable length, denoted x_n_) that is recognized by the extended PX domain of SNX5 and SNX6 (19, 21). Recent advances have revealed the identity of transmembrane cargoes that undergo sequence-dependent recognition by ESCPE-1 for sorting to the plasma membrane (19, 22), yet comparatively less is known about cargoes rerouted toward the TGN.

To identify the transmembrane proteins that undergo ESCPE-1–dependent retrograde transport, SILAC-based quantitative proteomics was used to compare HRP-TGN46–mediated biotinylation in Scramble (Scr) siRNA-treated and double SNX5 + SNX6 siRNA-treated HeLa cells (due to functional redundancy both SNX5 and SNX6 must be suppressed to perturb ESCPE-1–dependent trafficking) (17, 18). Retrograde trafficking of TGN46 has been demonstrated to be independent of ESCPE-1 (17, 18). Double SNX5 + SNX6 knockdown did not perturb HRP-TGN46 labeling at the whole cell lysate level (*SI Appendix*, Fig. S2*A*).

Of the previously established list of proteins biotinylated by HRP-TGN46, 46 proteins were significantly depleted (*P* < 0.05 Log_2_ fold change < −0.26) in the SNX5 + SNX6 siRNA HRP-TGN46–labeled proteome compared to Scramble siRNA-treated cells, of which 25 contained transmembrane-spanning domains (Fig. 2*B* and *SI Appendix*, Fig. S2*B* and Datasets S6–S8). STRING analysis identified a network of protein–protein interactions, including a cluster of proteins containing Neuropilin-1 (NRP1) alongside integrin-α5, integrin-β8, CD44, and the receptor tyrosine kinases MET and EPHA2 (Fig. 1*C*). Biological processes and molecular function categories pertaining to cellular adhesion and migration, transmembrane receptor kinase activity, and virus receptor activity were significantly enriched (Fig. 2*D* and Datasets S9–S11). Moreover, comparison of these proteins with published ESCPE-1 interactors and cell surface cargoes revealed a consistent enrichment of biological pathways (*SI Appendix*, Fig. S2*C*).

NRP1 is a coreceptor for a range of extracellular ligands, including members of the vascular endothelial growth factor (VEGF) and semaphorin families and was recently identified as a host factor that facilitates SARS-CoV-2 infection (23, 24). Moreover, NRP1 was also enriched in previously published interaction networks of SNX5, SNX6 (and the neuronal SNX6 paralogue SNX32), though its surface levels were unaffected by ESCPE-1 inactivating mutations (18, 19) (*SI Appendix*, Fig. S2*D*). Expression of an extracellular/luminally GFP-tagged murine NRP1 (GFP-Nrp1) revealed predominant localization to the plasma membrane and an internal population that colocalized with SNX1- and SNX6-decorated endosomes, and with the TGN markers TGN46, Golgin-97, and CI-MPR (Fig. 2 *E* and *F*). Moreover, we confirmed by immunofluorescence staining that the TGN-resident pool of endogenous NRP1 was reduced in HeLa cells lacking ESCPE-1 subunits (Fig. 2*G*).

**Fig. 3.**
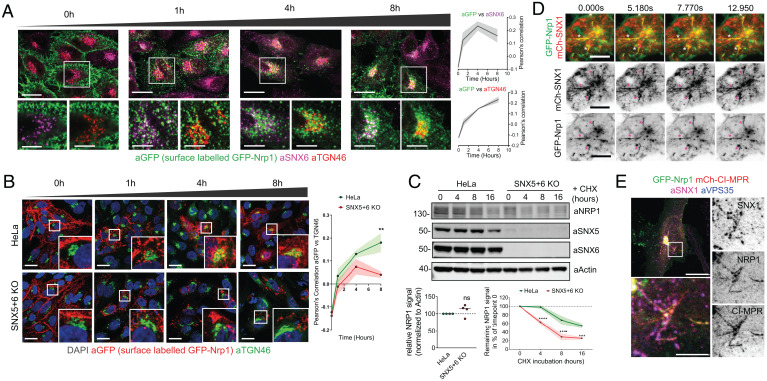
ESCPE-1 mediates tubular retrograde trafficking of NRP1. (*A*) Anti-GFP antibodies were bound to extracellular-facing GFP-Nrp1 in HeLa, followed by a surface uptake assay. At 0, 1, 4, and 8 h time points, cells were fixed and labeled with anti-SNX6 and anti-TGN46 antibodies; *n* = 3 independent repeats. (*B*) GFP-Nrp1 surface uptake assay in wild-type HeLa cells or SNX5 + 6 KO HeLa cells. Pearson’s correlation between anti-GFP and anti-TGN46 is measured over time. Colocalization was measured over *n* = 4 experiments with a two-way ANOVA and Šídák's multiple comparisons test, 0 h: *P* = 0.9897, 1 h: *P* = 0.6504, 4 h: *P* = 0.4805, 8 h: *P* = 0.0041. (Scale, 20 µm.) (*C*) Degradation assay in HeLa and HeLa SNX5 + SNX6 KO cells. Cells were incubated with 10 µg/mL cycloheximide and lysed at different time points as indicated. The band intensity of endogenous NRP1 was measured from *N* = 4 independent experiments using Odyssey software and normalized to wild-type HeLa levels. Steady-state levels of NRP1 were comparable between HeLa and SNX5 + 6 KO cells (*Left* graph). Two-tailed unpaired *t* test; *P* = 0.2803. The levels of NRP1 at different time points were compared using a two-way ANOVA and Šídák's multiple comparisons test (*Right* graph); 4 h: *P* < 0.0001, 8 h: *P* = < 0.0001, 16 h: *P* = 0.0008. (*D*) HeLa cells were cotransfected with GFP-Nrp1 and mCherry-SNX1 and live imaged after 24 h. Asterisk, examples of GFP-Nrp1 and SNX1 signals colocalizing in tubular profiles emanating from endosomes. (Scale bar, 10 µm.) (*E*) Confocal microscopy of HeLa cells cotransfected with mCherry-CI-MPR and GFP-Nrp1, costained with anti-SNX1 and anti-VPS35 antibodies. (Scale bar, 20 µm; zoom, 5 µm.) The bars, error bars, and circles represent the mean, SEM, and individual data points, respectively. **P <  0.01, ***P <  0.001, ****P <  0.0001; ns, not significant.

The extracellular/luminal GFP-tag on the Nrp1 construct was amenable for an antibody uptake assay to facilitate the chase of internalized GFP-Nrp1 from the cell surface into intracellular compartments following endocytosis (*SI Appendix*, Fig. S3*A*). At early time points GFP-Nrp1 colocalized with intracellular vesicles, a subpopulation of which were SNX6-positive endosomes, and progressively colocalized with TGN46 at later time points (Fig. 3*A* and *SI Appendix*, Fig. S3*B*). Three-dimensional (3D) reconstruction of the TGN46 signal revealed a population of internalized GFP-Nrp1 within the TGN (*SI Appendix*, Fig. S3*C*).

When we repeated the uptake assay in a previously described SNX5 + SNX6 double knockout (KO) HeLa cell line (19), GFP-Nrp1 showed a decreased rate of colocalization with TGN46 (Fig. 3*B*). Blocking of protein synthesis with cycloheximide (CHX) revealed an increased rate of endogenous NRP1 turnover in SNX5 + SNX6 KO cells, consistent with enhanced lysosomal degradation in the absence of retrograde endosomal sorting (Fig. 3*C*). Importantly no significant change of GFP-Nrp1 trafficking was observed at earlier time points, suggesting that internalization and early endocytic trafficking of GFP-Nrp1 is not altered in the SNX5 + SNX6 KO cells (Fig. 3*B*).

ESCPE-1 mediates retrograde endosomal trafficking by driving the biogenesis of cargo-enriched tubulovesicular membrane carriers that emanate from endosomes and couple to dynein/dynactin motor complexes for transport toward the TGN (1, 25–27). Live imaging of cells coexpressing GFP-Nrp1 and mCherry-SNX1 revealed enrichment of the receptor in tubular profiles emanating from SNX1 endosomes (Fig. 3*D* and *SI Appendix*, Fig. S3*D* and Movies S1 and S2). To assess whether these were indeed tubular carriers undergoing endosome-to-TGN trafficking, we coexpressed GFP-Nrp1 alongside a mCherry-CI-MPR construct, which is the prototypical ESCPE-1 retrograde cargo (17–19). Fluorescently tagged Nrp1 and CI-MPR colocalized in the same tubular profiles (Fig. 3*E* and *SI Appendix*, Fig. S3*E* and Movie S3), and endogenous SNX1 was found to decorate portions of these transport carriers (Fig. 3*E*). We conclude that ESCPE-1 mediates tubular-based sorting of NRP1 from endosomes to the TGN, through a similar pathway to that of CI-MPR.

**Fig. 4.**
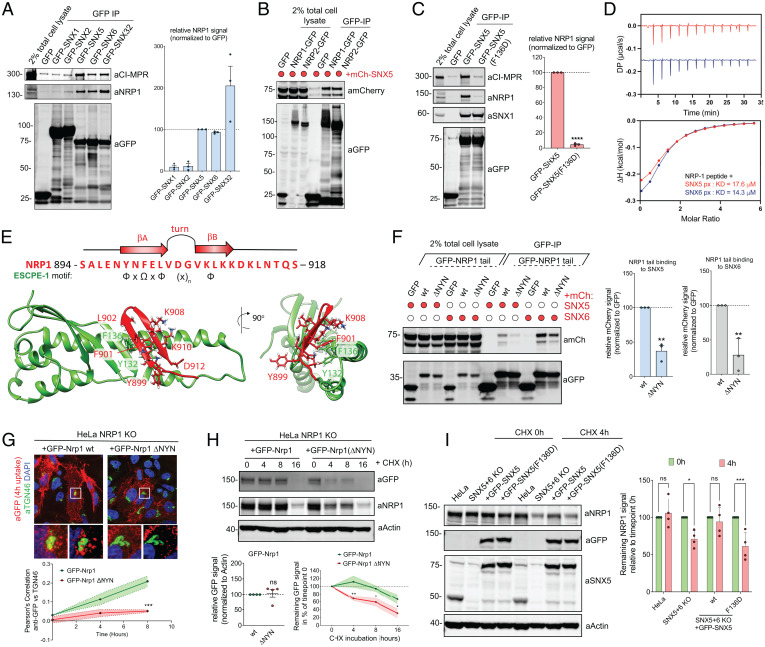
Molecular basis for the ESCPE-1 interaction with NRP1. (*A*) HEK293T cells were transfected to express GFP-tagged SNX1, SNXf2, SNX5, SNX6, and SNX32 and subjected to GFP-nanotrap (*n* = 3 independent experiments). (*B*) HEK293T cells were cotransfected to express GFP-tagged NRP1 or NRP2 and mCherry or mCherry-tagged SNX5 and subjected to GFP-nanotrap. The blot is representative of three independent experiments. (*C*) HEK293T cells were transfected to express GFP-tagged SNX5 or SNX5 F136D mutant and subjected to GFP-nanotrap (*n* = 3 independent experiments). Two-tailed unpaired *t* test; *P* < 0.0001. (*D*) SNX5 and SNX6 PX domain were titrated against the NRP1 peptide and binding was measured by ITC. *Top* shows the raw data and *Bottom* shows the integrated and normalized data fitted with a 1:1 binding model. The kDa values were measured over *n* = 3. (*E*, *Top*) Schematic of the NRP1 cytosolic domain sequence highlighting the residues that conform to the ESCPE-1 binding motif and that are predicted to fold into the beta-hairpin structure that engages the PX domain of SNX5. (*Bottom*) Molecular model of the cytosolic tail of NRP1 bound to the extended PX domain of SNX5. (*F*) HEK293T cells were cotransfected to express GFP-tagged NRP1 tail wild type or a ΔNYN mutant and mCherry-tagged SNX5 or SNX6 and subjected to GFP-nanotrap. SNX5 binding: two-tailed unpaired *t* test; *P* = 0.0013. SNX6 binding: two-tailed unpaired *t* test; *P* = 0.0064. (*G*) HeLaNRP1KO cells transfected with GFP-Nrp1 or GFP-Nrp1-ΔNYN mutant constructs were subjected to an antibody uptake assay for 0, 4, or 8 h. At the different time points cells were fixed and stained for TGN46, and anti-GFP colocalization was quantified in the GFP-positive subpopulation of cells (*n* = 3 independent experiments). Two-way ANOVA and Šídák's multiple comparisons test, 0 h *P* = 0.8060, 4 h *P* = 0.0736, 8 h *P* = 0.0004. (*H*) Degradation assay in HeLaNRP1KO cells transiently transfected with GFP-Nrp1 or GFP-Nrp1 ΔNYN mutant. At 48 h after transfection, cells were incubated with 10 µg/mL cycloheximide and lysed at different time points as indicated. The band intensity of GFP was measured from *N* = 4 independent experiments using Odyssey software. At steady state, GFP-Nrp1 or GFP-Nrp1 ΔNYN constructs expressed at comparable levels (*Left* graph), two-tailed unpaired *t* test; *P* = 0.8147. The levels of GFP-Nrp1 or GFP-Nrp1 ΔNYN at different time points were compared using a two-way ANOVA and Šídák's multiple comparisons test (*Right* graph); 4 h: *P* = 0.0091, 8 h: *P* = 0.0438, 16 h: *P* = 0.0397. (*I*) Degradation assay in HeLa, HeLa SNX5 + 6KO transduced to express GFP-SNX5 or the GFP-SNX5(F136D) mutant. Cells were incubated with 10 µg/mL cycloheximide and lysed at different time points as indicated. The band intensity of endogenous NRP1 was measured from *N* = 4 independent experiments using Odyssey software. In each cell line the degradation of NRP1 after 4 h was compared to the 0-h levels using a two-way ANOVA and Šídák's multiple comparisons test. HeLa: *P* = 0.9455; SNX5 + 6 KO: *P* = 0.0112; +GFP-SNX5: *P* = 0.9416; +GFP-SNX5(F136D): *P* = 0.0005. The bars, error bars, and circles represent the mean, SEM, and individual data points, respectively. *P <  0.05, **P <  0.01, ***P <  0.001, ****P <  0.0001; ns, not significant.

GFP-nanotrap of GFP-tagged ESCPE-1 subunits revealed that SNX5, SNX6, and SNX32 were able to immunoprecipitate endogenous NRP1, alongside CI-MPR (Fig. 4*A*). SNX1 and SNX2 were unable to coimmunoprecipitate CI-MPR and NRP1, consistent with the absence of the specialized cargo-binding extension within their PX domains (19). Moreover, NRP1 was also enriched in SILAC-based interactomes of SNX5, SNX6, and SNX32 (18) (*SI Appendix*, Fig. S4*A*). The interaction with SNX5 and SNX6 was also observed for the NRP1 homolog NRP2 (Fig. 4*B* and *SI Appendix*, Fig. S4 *B* and *C*). Conversely, GFP-tagged NRP1 tail immunoprecipitated mCherry-tagged SNX5 and SNX6, and all endogenous ESCPE-1 subunits (*SI Appendix*, Fig. S4 *D* and *E*).

We mapped NRP1 binding to the SNX5 PX domain by engineering a SNX1 chimeric construct where the SNX1 PX domain was replaced with that of SNX5; this successfully immunoprecipitated endogenous NRP1 (*SI Appendix*, Fig. S4*F*). Mutagenesis of the F136 residue within the extended SNX5 PX domain to aspartate prevented the interaction with NRP1, consistent with its inhibitory impact on CI-MPR binding (19) (Fig. 4*C*). The cytosolic tail of NRP1 contains a stretch of residues (894 to 918), conserved in NRP1 and NRP2, conforming to the ФxΩxФx_n_Ф motif for SNX5/SNX6 PX domain binding (*SI Appendix*, Fig. S4*G*). Isothermal titration calorimetry (ITC) showed that a synthetic peptide corresponding to these residues in NRP1 directly bound the PX domain of SNX5 and SNX6 with an affinity of 19.8 µM and 14.3 µM respectively, which is similar in strength to other known cargoes (19) (Fig. 4*D* and *SI Appendix*, Fig. S4*G*). Next, using structures of ФxΩxФx_n_Ф cargoes bound to the SNX5 PX domain (19), we generated a molecular model for the NRP1:SNX5 PX interaction. This was consistent with the NRP1 cytosolic tail folding into a beta hairpin that docked into the SNX5 PX domain (βA: 898-NYNFELV-904, loop: 905-DG-906, βB: 907-VKLKKD-912) (Fig. 4*E*).

To validate the model, we generated a panel of mutations targeting the residues within the predicted βA and βB strands in the GFP-tagged cytosolic tail of NRP1 (*SI Appendix*, Fig. S4*H*). Immunoprecipitation experiments revealed that the mutant forms of the NRP1 tail displayed a reduced interaction with mCherry-SNX6 (*SI Appendix*, Fig. S4 *I* and *J*). ITC experiments confirmed a dramatic loss of affinity of the NRP1 tail for the PX domain of SNX5 when the aromatic residues Y899 and F901 were mutated (*SI Appendix*, Fig. S4*K*). Furthermore, a triple deletion of the βA residues ^898^NYN^900^ produced the largest reduction in mCherry-SNX6 immunoprecipitation and endogenous SNX5 and SNX6 immunoprecipitation (Fig. 4*F*). Importantly, deletion of these residues did not affect association with GIPC1, the scaffolding protein that regulates NRP1 internalization through binding to a C-terminal PSD-95/Dlg/ZO-1 (PDZ)-binding motif in NRP1 (28–30) (*SI Appendix*, Fig. S4*L*).

We next compared the trafficking of GFP-Nrp1 and GFP-Nrp1 ΔNYN in previously characterized NRP1 KO HeLa cells (23). Although the total and surface levels of GFP-Nrp1 and GFP-Nrp1 ΔNYN Nrp1 were comparable, the retrograde trafficking of the mutant was reduced when compared to GFP-Nrp1 (Fig. 4*G* and *SI Appendix*, Fig. S4*M*) and resulted in the increased degradation of the mutant receptor (Fig. 4*H*). Consistently, in SNX5 + 6 double KO cells, GFP-tagged SNX5 rescued NRP1 turnover rate to wild-type (WT) levels, whereas rescue with GFP-SNX5(F136D), which does not immunoprecipitate NRP1, failed to do so (Fig. 4 *C* and *I*). These data establish that NRP1 possesses a canonical ESCPE-1 binding motif, and that direct interaction between NRP1 and ESCPE-1 is required for the correct retrograde trafficking of the internalized receptor.

**Fig. 5.**
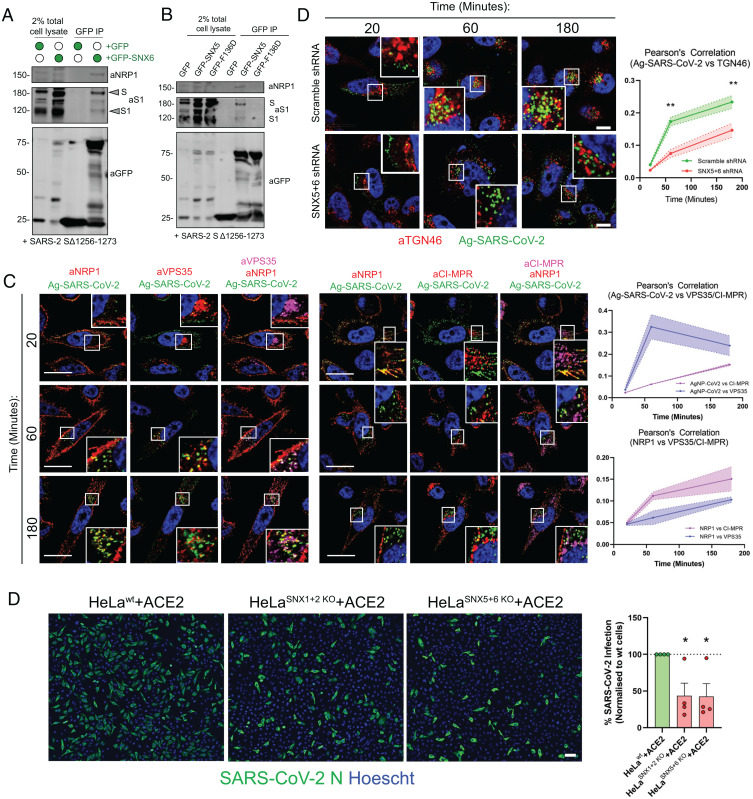
ESCPE-1 interacts with the SARS-CoV-2 spike protein through a cargo-selective mechanism and mediates trafficking of nanoparticles coated with the SARS-CoV-2 spike CendR motif. (*A*) HEK293T cells stably expressing SARS-CoV-2 S (SARS-2 SΔ1256 to 1273) were transfected to express GFP-tagged SNX6 or GFP and subjected to GFP-nanotrap. (*B*) HEK293T cells stably expressing the SARS-CoV-2 S gene (SARS-2 SΔ1256 to 1273) were transfected to express GFP-tagged SNX5 or SNX5(F136D) and subjected to GFP-nanotrap. (*C*) Ag-SARS-CoV-2 uptake assay in PPC-1 cells. Pearson’s correlation between Ag-SARS-CoV-2 and anti-VPS35 or anti-CI-MPR is measured over time using ImageJ software (*n* = 3 experiments). (Scale bar, 20 µm.) (*D*) Ag-SARS-CoV-2 uptake assay in Scramble shRNA PPC-1 cells or SNX5 + 6 shRNA PPC-1 cells. Pearson’s correlation between Ag-SARS-CoV-2 and anti-TGN46 is measured over time using ImageJ software (*n* = 3 experiments). Two-way ANOVA with Šídák's multiple comparisons test, *P* = 0.8154 (20 min), *P* = 0.0013 (60 min), *P* = 0.0034 (180 min). (Scale bar, 20 µm.) (*D*) HeLa^WT^ + ACE2, HeLa^SNX1 + 2 KO^ + ACE2 and HeLa^SNX5 + 6 KO^ + ACE2 cells were infected with SARS-CoV-2 and fixed at 6 hpi. Cells were stained for the SARS-CoV-2 N protein (green) and Hoescht (blue), and infection levels were quantified and normalized to HeLa^WT^ + ACE2 cells. (Scale bar, 50 µm.) (*n* = 4 independent experiments) One-way ANOVA with Dunnett’s multiple comparisons test: HeLa^WT^ + ACE2 vs. HeLa^SNX1 + 2 KO^ + ACE2 *P* = 0.0364; HeLa^WT^ + ACE2 vs. HeLa^SNX5 + 6 KO^ + ACE2 *P* = 0.0338. *P < 0.05, **P < 0.01.

We recently established that the extracellular b1 domain of NRP1 directly associates with a multibasic C-terminal motif (termed the C-end rule motif) (31) in the furin-processed SARS-CoV-2 spike S1 subunit to facilitate infectivity (23). Therefore, we investigated whether ESCPE-1 could associate with the SARS-CoV-2 spike (S) protein via NRP1. In HEK293T cells stably expressing a C-terminally truncated SARS-CoV-2 S gene (SARS-2 SΔ1256 to 1273), GFP-SNX5 and GFP-SNX6 coimmunoprecipitated bands corresponding to S1 and uncleaved S alongside endogenous NRP1 (Fig. 5 *A* and *B*). SARS-CoV-2 S does not contain a putative ФxΩxФx_n_Ф motif within its cytosolic tail, suggesting that coimmunoprecipitation likely occurs through an intermediate protein. Importantly, the association between SNX5 and spike was abrogated by the SNX5(F136D) mutation incapable of binding NRP1, consistent with spike binding being mediated through an NRP1-dependent mechanism (Fig. 5*B*).

To investigate whether the interaction between NRP1 and ESCPE-1 may regulate retrograde trafficking of internalized NRP1-bound viral particles, we engineered a minimal system comprising approximately coronavirion-sized (80 nm) silver nanoparticles coated with the C-end rule (CendR) peptide motif of the SARS-CoV-2 S1 protein (Ag-SARS-CoV-2, sequence: TNSPRRAR, original isolate) (23, 24). Silver nanoparticles decorated with CendR sequences specifically bind NRP1 at the cell surface and are internalized through an NRP1-dependent mechanism (24, 31, 32). Accordingly, NRP1-expressing PPC-1 cells incubated with Ag-SARS-CoV-2 demonstrated colocalization with NRP1 at the cell surface at early time points (Fig. 5*C*). Adherence at the cell surface was followed by internalization of nanoparticles and transit toward the perinuclear region, visualized by early colocalization with the endosomal marker VPS35 and accumulating colocalization with CI-MPR and TGN46 over 3 h (Fig. 5 *C* and *D*). This phenomenon was ESCPE-1 dependent, as SNX5 + SNX6 knockdown PPC-1 cells demonstrated a significant decrease in Ag-SARS-CoV-2 colocalization with TGN46 and Golgin-97 (Fig. 5*D* and *SI Appendix*, Fig. S5*A*). These data suggest that ESCPE-1 can govern the endosomal trafficking of internalized NRP1 ligands, raising the possibility of a role for the complex in SARS-CoV-2 infection (*SI Appendix*, Fig. S5*B*).

To assess the role of ESCPE-1 in SARS-CoV-2 infection, we transduced SNX1 + SNX2 double KO and SNX5 + SNX6 double KO HeLa cell lines with ACE2 (*SI Appendix*, Fig. S5*B*). We performed infection assays using the SARS-CoV-2 isolate SARS-CoV-2/human/Liverpool/REMRQ001/2020 (23) and fixed cells at 6 h postinfection (hpi). As detected by nucleoprotein (N) expression, infectivity was reduced in HeLa^SNX1+SNX2 KO^ and HeLa^SNX5+SNX6 KO^ cells relative to wild-type control cells, at both time points (Fig. 5*D*). This reduction was not due to decreased expression of the SARS-CoV-2 receptor ACE2 or NRP1 at the cell surface as shown by their unaltered surface biotinylation (*SI Appendix*, Fig. S5*C*) (33). Taken together, these data suggest a proviral role for ESCPE-1 in SARS-CoV-2 infection through the regulation of NRP1 trafficking.

## Discussion

We and others recently demonstrated that SARS-CoV-2 directly interacts with NRP1 at the cell surface to enhance cellular infection (23, 24). In the present study, through the development of an unbiased proteomic screen to discover retrograde endosomal cargoes, we identified a range of potential transmembrane cargoes for the evolutionarily conserved ESCPE-1 complex. From this screen, we validate NRP1 as an interactor of ESCPE-1. ESCPE-1 directly engages the cytosolic tail of NRP1 in a sequence-dependent manner and mediates tubulovesicular endosomal sorting of NRP1 to the TGN. Additionally, we identify a wider functional network of transmembrane proteins perturbed by SNX5 + SNX6 depletion, including integrin-α5 and integrin-β8, and the receptor tyrosine kinases MET and EPHA2, raising the possibility that ESCPE-1–mediated retrograde sorting may play a role in directional cell migration and signaling (34–36). NRP1 has a reported role in mediating integrin internalization and trafficking (29, 37) and associates with receptor tyrosine kinases, including the MET receptor to modulate their signaling outputs (38, 39). Our study thus provides mechanistic insight into the previously described role of retrograde endosomal sorting in cell migration (10, 36).

Considering the recent identification of NRP1 as a SARS-CoV-2 host factor, the mechanistic basis of its intracellular trafficking by ESCPE-1 opens interesting avenues for future investigation. SARS-CoV-2 exhibits two distinct cellular entry pathways: either direct fusion with the plasma membrane or internalization into endosomal compartments (40). It is presently unknown whether the SARS-CoV-2 virions that are internalized through endocytosis can hijack NRP1- and ESCPE-1–dependent endocytic trafficking to subvert innate cellular defenses, or whether the spike protein alone undergoes this trafficking step. We demonstrate that ESCPE-1 coimmunoprecipitates the SARS-CoV-2 spike through a sequence-dependent mechanism likely dependent on NRP1. Furthermore, CRISPR-Cas9–mediated deletion of ESCPE-1 subunits attenuated SARS-CoV-2 infection in HeLa + ACE2 cells, suggesting a role for the complex in the infection mechanism. Engineered nanoparticles displaying the CendR motif of SARS-CoV-2 spike hijacked ESCPE-1–dependent retrograde trafficking. ESCPE-1 couples to the dynein–dynactin complex to provide a mechanical pulling force that aids the biogenesis of tubular endosomal carriers that traffic toward the perinuclear region (25–27). This process may conceivably provide additional energy input and membrane tension to facilitate the virus endosomal uncoating, as seen in influenza A virus (41, 42). Indeed, modeling of the SARS-CoV-2 spike protein binding to NRP1 and ACE2 has suggested that NRP1 facilitates S1/S2 separation, a prerequisite for membrane fusion (43).

Interestingly, additional pathogens hijack ESCPE-1 cargo recognition to promote intracellular survival (2, 3, 44), and SNX5 has also recently been identified as a key regulator of innate cellular immunity against a range of viruses (45). Genomewide CRISPR screens and biochemical studies have identified multiple endosomal sorting machineries that facilitate SARS-CoV-2 infection, including components of the retromer, retriever and COMMD/CCDC22/CCDC93 (CCC) complexes (46–51). Here, we suggest that ESCPE-1 sequence-dependent cargo sorting also plays a role in regulating the endosomal dynamics exploited during SARS-CoV-2 infection, potentially expanding the scope of pathogens that can exploit this complex.

A fascinating and impactful emerging theme is that the NRP1 pathway may influence infection by a wide range of viruses through the recognition of CendR motifs on viral glycoproteins (31, 52). Our findings therefore highlight the possibility that multiple viruses converge upon a NRP1- and ESCPE-1–dependent intracellular trafficking pathway within the endosomal network. We previously demonstrated that pharmacological inhibition of the SARS-CoV-2–NRP1 interaction limits infection in cell culture (23). Future work will be required to appreciate the importance of NRP1 trafficking in SARS-CoV-2 biology and the wider range of pathogens that exploit this receptor to mediate infectivity.

## Materials and Methods

### Antibodies.

Antibodies used in this study were as follows: GFP (clones 7.1 and 13.1; 11814460001; Roche) (1:2,000 for Western blot (WB), 1:400 for immunofluorescence (IF)), SNX1 (clone 51/SNX1; 611482; BD) (1:1,000 for WB, 1:200 for IF), SNX2 (clone 13/SNX2; 5345661; BD) (1:1,000 for WB, 1:200 for IF), SNX6 (clone d-5,365965; Santa Cruz Biotechnology, Inc.) (1:1,000 for WB, 1:200 for IF), SNX5 (clone EPR14358; ab180520; Abcam) (WB 1:500), β-actin (A1978; Sigma-Aldrich) (1:2,000 for WB), ITGA5 (610633, BD) (1:1,000 for WB); VPS35 (97545; Abcam) (IF 1:200), GFP (GTX20290; GeneTex) (WB 1:2,000), EEA1 (C45B10; Cell Signaling Technologies) (IF 1:200), LAMP1 (clone H4A3, AB2296838; DSHB) (IF 1:200) Cathepsin D (21327-1-AP; Proteintech) (WB 1:1,000), Calnexin (ab22595; Abcam) (WB 1:1,000), TGN46 (AHP500G, Bio-Rad) (IF 1:400), Golgin-97 (clone CDF4; A-21270; Thermo Fischer) (IF 1:200), HA tag (66006-1-Ig, Proteintech) (WB 1:1,000), NCAD (clone 13A0; 14215; Cell Signaling) (WB 1:1,000), Tom20 (clone 29; 612278; BD Biosciences), GALNT2 (ab102650; Abcam) (WB 1:1,000), NRP1 (clone EPR3113; ab81321; Abcam) (WB 1:1,000, IF 1:200), NRP1 b1b2 domain (23, 24) (clone 3E7.1) (IF 1:200), Tubulin (ab6046; Abcam) (WB 1:2,000), SARS-CoV-2 spike (40592-T62, Sino Biologicals) (WB 1:1,000) SARS Nucleocapsid (200-401-A50, Rockland) (IF 1:2,000).

### Cell Culture and Transfection.

HeLa and HEK293T cell lines were sourced from the American Type Culture Collection (ATCC). Authentication was from the ATCC. PPC-1 human primary prostate cancer cells were obtained from Erkki Ruoslahti’s laboratory at Cancer Research Center, Sanford Burnham-Prebys Medical Discovery Institute, La Jolla, CA. Cells were grown in Dulbecco's Modified Eagle Medium (DMEM) (Sigma-Aldrich) supplemented with 10% (vol/vol) FCS (Sigma-Aldrich) and penicillin/streptomycin (Gibco) and grown under standard conditions. FuGENE HD (Promega) was used for transient transfection of DNA according to the manufacturer’s instructions. Cycloheximide was used to block protein synthesis. Cycloheximide (C7698; Sigma-Aldrich) at 10 µg/mL was added to the cells for the indicated time points. The SNX1 + SNX2 and SNX5 + SNX6 knockout clonal cell lines, and HeLa^WT^ + ACE2 and HeLa^NRP1 KO^ + ACE2 cell lines used in this study were characterized previously (18, 19, 23). For GFP-based immunoprecipitations, HEK293T cells were transfected with GFP constructs using polyethylenimine (Sigma-Aldrich) and expression was allowed for 48 h. For siRNA-based knockdown, cells were first reverse transfected using DharmaFECT (GE Healthcare) and then transfected again with HiPerFect (QIAGEN) 24 h later according to the manufacturer’s instructions. At 48 h after the second transfection, cells were lysed or fixed and stained. SNX5 + 6 suppression was performed using a combination of the following oligonucleotides against SNX5 (sequence 5′-CUACGAAGCCCGACUUUGA-3′) and SNX6 (sequence 5′-UAAAUCAGCAGAUGGAGUA-3′). To perform knockdowns in PPC-1 cells, pLKO.1-puro-CMV-tGFP plasmids targeting SNX5 (5′-ACTATTACAATAGGATCAAAG-3′, 5′-CTGAGTATCTCGCTGTGTTTA-3′), and SNX6 (5′-AGTAAAGGATGTAGATGATTT-3′, 5′-GCCGAAACTTCCCAACAATTA-3′) (Sigma-Aldrich) were lentivirally transduced, and GFP-positive cells were quantified as knockdowns. HeLa cells were transduced with lentiviruses, with constructs cloned into pXLG3 or pLVX vectors, to produce stably expressing cell lines. Following transduction, transduced cells were selected with puromycin or blasticidin accordingly.

### Immunoprecipitation and Quantitative Western Blot Analysis.

For Western blotting, cells were lysed in phosphate buffered saline (PBS) with 1% (vol/vol) Triton X-100 and protease inhibitor mixture. The protein concentration was determined with a bicinchoninic acid (BCA) assay kit (Thermo Fisher Scientific), and equal amounts were resolved on NuPAGE 4 to 12% precast gels (Invitrogen). Blotting was performed onto polyvinylidene fluoride membranes (Immobilon-FL; EMD Millipore) followed by detection using the Odyssey infrared scanning system (LI-COR Biosciences). For GFP-based immunoprecipitations, HEK293T cells were lysed 48 h after transfection in immunoprecipitation buffer (50 mM Tris⋅HCl, 0.5% (vol/vol) Nonidet P-40, and Roche protease inhibitor mixture) and subjected to GFP trap (ChromoTek). Immunoblotting was performed using standard procedures. Detection was performed on an Odyssey infrared scanning system (LI-COR Biosciences) using fluorescently labeled secondary antibodies.

### HRP-TGN46 Proximity Biotinylation.

The methodology for HRP-TGN46 biotinylation is adapted from the APEX2 biotinylation protocol outlined in ref. 15. The 10 × 10^6^ HRP-TGN46–expressing cells were seeded in a 15-cm plate the day before biotinylation. The next day, cells were incubated in DMEM media supplemented with 500 μM BP and incubated for 30 min at 37 °C. H_2_O_2_ was added at a final concentration of 1 mM and distributed by rocking the cell plate. After 45 s of H_2_O_2_ incubation, the media was removed and replaced with ice-cold, freshly prepared quencher solution consisting of 1 mM sodium ascorbate, 500 μM (±)-6-hydroxy-2,5,7,8-tetramethylchromane-2-carboxylic acid (Trolox; Sigma-Aldrich, 238813) and 1 mM sodium azide in PBS to ensure that the biotinylation reaction does not proceed beyond 1 min. The quencher solution was left for 1 min, then discarded, and this washing process was repeated five times. Following washes, cells were lysed in RIPA buffer (150 mM NaCl, 0.1% [vol/vol] SDS, 0.5% [wt/vol] sodium deoxycholate, 1% [vol/vol] Triton X-100, protease inhibitor mixture, 50 mM Tris⋅HCl, pH 7.5) and lysates spun at 20,000 × *g* for 10 min at 4 °C. The 50-μL aliquots of streptavidin beads were prepared and washed three times in RIPA buffer, centrifuging beads between washes at 350 × *g*. The cell lysates were mixed with the streptavidin beads and rotated for 1 h at 4 °C.

After incubation, streptavidin beads were centrifuged and the supernatant containing unbound proteins was removed. The beads were washed seven times (twice in RIPA buffer, once with 1 M KCl, once with 0.1 M Na_2_CO_3_, once with 2 M urea 10 mM Tris⋅HCl pH 8.0, and twice again with RIPA buffer). All buffers were kept ice cold throughout the process. After the final wash step, all supernatant was aspirated off and beads were resuspended in 3× NuPAGE sample buffer supplemented with 2.5% β-mercaptoethanol, 2 mM free biotin, and 20 mM dithiothreitol (DTT).

### Biotinylation of Cell Surface Proteins.

For surface biotinylation experiments, fresh sulfo-NHS-SS biotin (Thermo Scientifics, #21217) was dissolved in ice-cold PBS at pH 7.8 at a final concentration of 0.2 mg/mL. Cells were washed twice in ice-cold PBS and placed on ice to slow down the endocytic pathway. Next, cells were incubated with the biotinylation reagent for 30 min at 4 °C followed by incubation in tris-buffered saline (TBS) for 10 min to quench the unbound biotin. The cells were then lysed in lysis buffer and subjected to streptavidin bead–based affinity isolation (GE Healthcare).

### Immunofluorescence Staining.

Cells were fixed in 4% (vol/vol) paraformaldehyde (PFA) for 20 min and washed three times in PBS and permeabilized with 0.1% (vol/vol) Triton X-100. Fixed cells were blocked in 1% (wt/vol) bovine serum albumin (BSA) and incubated in primary antibody and respective secondary antibody (Alexa Fluor; Thermo Fisher Scientific) in 1% (wt/vol) BSA. Biotinylated proteins were labeled with 0.5 μg/mL Alexa Fluor 568-conjugated streptavidin. For uptake assays, HeLa cells were transfected with GFP-Nrp1 constructs. At 24 h after transfection, cells were incubated with anti-GFP antibody on ice for 30 min, then returned to 37 °C media. GFP-Nrp1 trafficking was followed for 0 h, 1 h, 4 h, and 8 h prior to fixation. The retrograde transport to the TGN was assayed through measuring colocalization of anti-GFP signal with the TGN marker TGN46.

### Electron Microscopy.

A total of 100,000 HRP-TGN46–expressing HeLa cells were seeded in glass bottom 35-mm dishes (MatTek, P35G-1.5-14-CGRD) the day before sample processing. Cells were fixed in solution of 2.5% glutaraldehyde, 3 mM CaCl_2_, 0.1 M cacodylate buffer, pH 7.4, for 5 min at room temperature, then 1 h on ice. All solutions and incubation steps from this point onwards until the embedding stage were kept ice cold. Samples were washed five times in 0.1 M cacodylate buffer, leaving each wash on ice for 2 min. Samples were incubated in a quenching buffer of 20 mM glycine and 0.1 M cacodylate for 5 min, then washed in 0.1 M cacodylate buffer an additional five times, at 2 min per wash. A 1× DAB, 10 mM H_2_O_2_ solution was assembled by dissolving 50 mg of DAB in 10 mL of HCl, then diluting 1 mL of this solution in 9 mL of 0.1 M cacodylate and 10 µL of 30% (wt/wt) H_2_O_2_.

To obtain differential interference contrast (DIC) images of DAB polymerization, a Leica DM IRBE inverted epifluorescence microscope (Leica Microsystems) was used. An initial picture was taken prior to DAB labeling. The 0.1 M cacodylate washing buffer was removed and replaced with the 1 mL of 1× DAB 10 mM H_2_O_2_ solution. DAB polymerization was observed through the eyepiece in real time, and DIC images were taken. The reaction was stopped by removing the solution and washing five times with 0.1 M cacodylate buffer, at 2 min per wash. The final wash was removed, and cells were incubated in 1% OsO_4_ for 30 min. This solution was removed, then samples were washed with distilled water three times, at 1 min per wash. Finally, samples were incubated overnight in a filtered solution of 2% uranyl acetate in distilled water at 4 °C.

The next day, samples were dehydrated by sequential 3-min incubations of 20%, 50%, 70%, 90%, 100%, 100% ice-cold ethanol, followed by a final 3-min wash of 100% ethanol at room temperature. The final ethanol wash was removed and EPON resin (TAAB, T031) was poured onto the samples and then samples were left rocking for 3 h. The resin was discarded and then fresh EPON resin was poured onto samples. The samples were incubated at 60 °C overnight to set the resin. A small volume of fresh EPON was poured into the middle of the sample and then used to adhere an EPON stub. The sample was returned to 60 °C for an additional 24 h. The coverslip was removed from the resin by sequential plunging into liquid nitrogen and boiling water. The embedded resin was cut into <70-nm slices for transmission electron microscopy using a Leica UC6 cryo ultra microtome (Leica Microsystems). Samples were imaged on a FEI Tecnai 12 120 kV BioTwin Spirit transmission electron microscope (FEI Company).

### Image Acquisition and Image Analysis.

Microscopy images were collected with a confocal laser-scanning microscope (SP5 AOBS; Leica Microsystems) attached to an inverted epifluorescence microscope (DMI6000; Thermo Fisher Scientific). A 63× 1.4 numerical aperture (NA) oil immersion objective (Plan Apochromat BL; Leica Biosystems) and the standard SP5 system acquisition software and detector were used. For stimulated emission depletion (STED) microscopy, images were taken using a Leica SP8 confocal laser scanning microscope attached to a Leica DMi8 inverted epifluorescence microscope (Leica Microsystems) with a 63× HC PL APO CS2 oil immersion lens, NA 1.4 (Leica Microsystems, 506351). Images were captured at room temperature as z stacks with photomultiplier tube detectors with a photocathode made of gallium-arsenide-phosphide (Leica Microsystems) for collecting light emission. Images were captured using Application Suite AF software (version 2.7.3.9723; Leica Microsystems) and then analyzed with the Volocity 6.3 software (PerkinElmer) or ImageJ. For live cell imaging, cells were seeded in dishes (MatTek) in prewarmed CO_2_-independent media. Cells were imaged with a confocal laser scanning microscope (SP8 AOBS; Leica Microsystems) attached to a DMI6000 inverted epifluorescence microscope with an HCX Plan Apochromat lambda blue 63× 1.4 NA oil objective. Images were captured at 37 °C, and “adaptive focus control” was used to correct focus drift during time courses.

### Statistics and Reproducibility.

All quantified Western blot are the mean of at least three independent experiments. Statistical analyses were performed using GraphPad Prism 9. Graphs represent means and SEM. For all statistical tests, *P* < 0.05 was considered significant and is indicated by asterisks.

### SILAC-Based Proteomics.

HeLa cells expressing HRP-TGN46 were cultured for at least six doublings in three different isotopically labeled media compositions: R_0_K_0_ (light), R_6_K_4_ (medium), and R_10_K_8_ (heavy) (Silantes). After performing HRP-TGN46 biotinylation and streptavidin affinity purification, the streptavidin beads corresponding to different SILAC conditions were pooled together prior to washing steps. Biotinylated proteins were eluted in a volume of 40 μL 3× NuPAGE sample buffer supplemented with 2.5% β-mercaptoethanol, 2 mM free biotin, and 20 mM DTT. The eluate was loaded onto a gel, and proteins were resolved by sodium dodecyl sulfate–polyacrylamide gel electrophoresis (SDS-PAGE), then visualized by staining with SimplyBlue SafeStain (Thermo Fisher, LC6060). The gel lane was cut into 10 equal slices, which were subjected to in-gel tryptic digestion and the resulting peptides analyzed by nano-liquid chromatography tandem mass spectrometry (LC-MS/MS) using an Orbitrap Fusion Tribrid mass spectrometer (Thermo Fisher Scientific).

The raw data files were processed and quantified using Proteome Discoverer software v2.1 (Thermo Fisher Scientific) and searched against the UniProt Human database using the SEQUEST HT algorithm. Peptide precursor mass tolerance was set at 10 ppm, and MS/MS tolerance was set at 0.6 Da. Search criteria included carbamidomethylation of cysteine as a fixed modification and oxidation of methionine, appropriate SILAC labels, and the addition of biotin-phenol (+361.146 Da) to tyrosine as variable modifications. Searches were performed with full tryptic digestion and a maximum of four missed cleavages were allowed. The reverse database search option was enabled and all data were filtered to satisfy false discovery rate (FDR) of 5%.

For statistical analysis of differential protein abundance between conditions, standard *t* tests were used. Volcano plots were plotted using Orange software (University of Ljubljana) or VolcanoseR (53). For generation of the HRP-TGN46–labeled proteome, proteins that were only identified in the HRP-TGN46 biotinylation condition in ≥4 out of 5 repeats and were not identified in negative control conditions and thus could not be statistically analyzed, were assumed to be significant hits (Dataset S2). Furthermore, 10 proteins significantly enriched in the heavy SILAC condition (HRP-TGN46 + BP − H_2_O_2_) relative to the light SILAC condition (WT HeLa + BP + H_2_O_2_) were identified and removed from downstream analyses.

Gene ontology analysis was performed using the PANTHER classification system (54) and Metascape (55). Protein identifications for enriched or depleted proteins were compared against the total human genome. Gene ontology terms, falling under the categories of “cellular component,” “biological process,” or “molecular function” that were significantly enriched or depleted relative to the expected number for the sample size of proteins were identified by a Fisher’s exact test with the Bonferroni correction for multiple testing by PANTHER software.

### Recombinant Protein Expression and Purification.

The expression plasmid of pGEX-4T-2 containing GST-tagged SNX5 and SNX6 PX domain constructs were transformed into *Escherichia coli* BL 21 (DE3) cells and plated on lysogeny-broth (LB) agar plates supplemented with ampicillin (0.1 mg/mL). Single colony was then used to inoculate 50 mL of the LB medium containing ampicillin and the culture was grown overnight at 37 °C with shaking at 180 rpm. The following day, 1 L of LB medium containing the antibiotic ampicillin (0.1 mg/mL) was inoculated using 10 mL of the overnight culture. Cells were then grown at 37 °C with shaking at 180 rpm to an optical density of 0.8 to 0.9 at 600 nm and the protein expression was induced by adding 0.5 mM (isopropyl-b-D-thiogalactopyranoside (IPTG). Expression cultures were incubated at 20 °C overnight and the cells were harvested the next day by centrifugation at 4,000 rpm for 15 min using Beckman rotor JLA 8.100. Cell pellets were then resuspended in 20 mL (for cell pellet from 1 L) of lysis buffer (50 mM Hepes, pH 7.5, 500 mM NaCl, 5% glycerol, benzamidine [0.1 mg/mL], and Dnase [0.1 mg/mL]). Resuspended cells were lysed by using the cell disrupter (Constant Systems, LTD, UK, TS-Series) and the soluble fraction containing the protein was separated from cell debris by centrifugation at 18,000 rpm for 30 min at 4 °C. The soluble fraction was first purified by affinity chromatography using glutathione Sepharose 4B resin (GE Healthcare) and the GST tag was cleaved by incubating the protein with thrombin (Sigma-Aldrich) overnight at 4 °C. The next day the protein was eluted using 50 mM Hepes, pH 7.5, 200 mM NaCl. The eluted protein was then concentrated and further purified by gel filtration chromatography (Superdex 75 (16/600), GE Healthcare) using 50 mM Hepes, pH 7.5, 200 mM NaCl, 0.5 mM tri(2-carboxyethyl)phosphine (TCEP) and the fractions corresponding to SNX5/SNX6 PX were analyzed by SDS-PAGE.

### ITC.

ITC experiments were carried out by using Microcal ITC200 instrument (Malvern) at 298 K. The NRP1 peptide was dissolved in the same buffer (50 mM Hepes, pH 7.5, 200 mM NaCl, 0.5 mM TCEP) as the proteins to avoid buffer mismatch. A total of 25 mM SNX5/6 PX was then titrated with 1 mM NRP1 peptide using a series of 13 injections of 3.22 mL each with 180-s intervals. The dissociation constant, *K*_d_ (1/*K*_a_), enthalpy of binding (Δ*H*), and the stoichiometry of the binding reaction (N) were calculated by fitting the data to a single site binding model using MicroCal PEAQ-ITC software. Experiments were performed in triplicate to check for reproducibility and the average values of the three experiments are reported.

### SNX5-NRP1 Modeling and Molecular Dynamics.

The model for the SNX5-NRP1 complex was built based on the 6n5z.pdb structure according to the method outlined in *SI Appendix*. Twenty nanosecond atomistic dynamic simulations of the modeled complexes were carried out using the amber99sb-ildn forcefield in TIP3P waters and GROMACS (56) (2019.2) according to the method described recently (57).

### Silver Nanoparticles Uptake Assay.

Silver nanoparticles labeled with the dye CF555 and functionalized with the biotinylated CendR peptide of the SARS-CoV-2 S1 protein (biotin-Ahx-TNSPRRAR; Ahx, aminohexanoic acid) were prepared as previously described (58, 59). The peptide was purchased from TAG.

PPC-1 cells (10^5^ cells) were seeded onto noncoated coverslips (12-mm diameter, Marienfeld Superior; Paul Marienfeld GmbH & Co.) in a 24-well plate and cultured for 24 h. The cells were incubated with the mouse monoclonal anti-human NRP1 antibody (clone 3E7.1) at 4 °C for 30 min, washed with cold medium and, subsequently incubated with the AgNPs (0.3 nM in DMEM with 10% fetal bovine serum (FBS)) at 37 °C. Cells were washed, treated with the etching solution to remove the noninternalized AgNPs [1 mM of K_3_Fe(CN)_6_ and Na_2_S_2_O_3_ in PBS] at room temperature for 5 min and washed with PBS. For immunosfluorescence staining, cells were fixed in 4% (vol/vol) PFA for 10 min, washed three times in PBS, and permeabilized with 0.2% (vol/vol) Triton X-100. Fixed cells were blocked in blocking buffer containing 5% BSA (wt/vol), 5% FBS (vol/vol), and 5% goat serum (vol/vol) in PBS with 0.05% Tween-20 (PBST), and incubated in primary antibody and respective secondary antibody (Alexa Fluor; Thermo Fisher Scientific) in blocking buffer diluted 1:5 with PBST. Cells were counterstained with DAPI and visualized using a confocal microscope FV1200MPE (Olympus) equipped with the UPlanSApo 60×/1.35 NA objective (Olympus). The images were analyzed using Olympus FluoView ver.4.2a Viewer software. The colocalization of the AgNPs with the different markers was quantified using ImageJ software.

### SARS-CoV-2 Infection Studies.

For SARS-CoV-2 infection studies cells were grown in clear 96-well microplates (Greiner Bio-One). Cells were infected with the SARS-CoV-2/human/Liverpool/REMRQ001/2020 isolate in MEM, supplemented with 2% FBS and 0.1 mM NEAA for 6 h at 37 °C. After the appropriate incubation time the cells were fixed with 4% PFA in PBS and washed with PBS. Subsequently, cells were permeabilized with 0.1% Triton-X in PBS, 1% BSA, and blocked with PBS, 1% BSA. The cells were then stained with a polyclonal rabbit anti-SARS Nucleoprotein (N) antibody (1:2,000) (Rockland) for 1 h. After a washing step the cells were further stained with an anti-rabbit AF647-labeled secondary antibody and Hoechst (1:10,000) (Thermo Fisher Scientific) for 30 min. Following another wash step with PBS, infection was quantified measuring SARS-*N*-positive cells using an automated high-content spinning-disk microscope CQ1 (Confocal Quantitative Image 130 Cytometer).

## Supplementary Material

Supplementary File

Supplementary File

Supplementary File

Supplementary File

Supplementary File

Supplementary File

Supplementary File

Supplementary File

Supplementary File

Supplementary File

Supplementary File

Supplementary File

Supplementary File

Supplementary File

Supplementary File

## Data Availability

All study data are included in the article and/or supporting information. Data have not been deposited in a publicly accessible database, but are available as supplementary datasets.
