# Supplementary material for: ESCPE-1 mediates retrograde endosomal sorting of the SARS-CoV-2 host factor Neuropilin-1
Source: Proc Natl Acad Sci U S A. 2022 Jun 13;119(25):e2201980119. doi: 10.1073/pnas.2201980119 (PMC9231623; doi:10.1073/pnas.2201980119)
Supplement: Supplementary File [file pnas.2201980119.sapp.pdf]

PNAS

Supplementary Information for

ESCPE-1 Mediates Retrograde Endosomal Sorting of the SARS-CoV-2 Host Factor Neuropilin-1

Boris Simonetti[1,*,‡], James L. Daly[1,*], Lorena Simón-Gracia[2], Katja Klein[3], Saroja Weeratunga[4], Carlos Antón-Plágaro[1], Allan Tobi[2], Lorna Hodgson[1], Philip Lewis[5], Kate J. Heesom[5], Deborah K. Shoemark[6], Andrew D. Davidson[3], Brett M. Collins[4], Tambet Teesalu[2], Yohei Yamauchi[3], Peter J. Cullen[1,‡].

*These authors contributed equally to this work

‡Corresponding author: bsimonetti@hotmail.com (B.S.); pete.cullen@bristol.ac.uk (P.J.C.)

This PDF file includes:

Supplementary text
Figures S1 to S5
Legends for Movies S1 to S3
Legends for Datasets S1 to S11
SI References

Other supplementary materials for this manuscript include the following:

Movies S1 to S3
Datasets S1 to S11

Supplementary Information Text:
Modelling the SNX5-NRP1 Complex.

The aim was to generate any models of the NRP1 cytosolic tail sequence with a beta hairpin that could be mapped onto crystal structures of beta hairpins bound to SNX5 (pdb codes 6n5X, 6n5Y, 6n5Z).

Using the NRP1 cytosolic tail sequence:

YCACWHNGMSERNLSALENYNFELVDGVKLKKDKLNTQSTYSEA.

A sequence similarity search in the pdb (1) for regions of similarity, produced nothing. HHPRED (1) and Modeller (2) failed because there were no suitably similar homologous structures on which to build a model. The RAFT (3) software was used to generate folds ab initio, but this only produced all helix-coiled-helix structures. Itasser (4), which combines homology and ab initio methods, produced templates with some stretches of beta but none had a convincing hairpin.

Returning to the 6n5z structure in the pdb a Clustal Omega (5) alignment of the NRP1 residues region known to bind SNX-5 with those of the semaphorin-4C residues that bind to SNX-5 in the crystal structure 6n5z.pdb.

>NRP1_SNX5-binding-residues.

SALENYNFELVDGVKLKKDKLNTQ

>SEMA4C_ SNX5-binding-residues.

NWDPVGYYYSDGSLKIVP

CLUSTAL O(1.2.4) multiple sequence alignment

```
NRP1_bit. SALENYNFELVDGV-KLKKDKLNTQ 24
SEMA4C.   -NWDPVGYYYSDGSLKIVP       18
             +   +   **   +
```

Although not too promising, the hairpin DGs did align with the VDGV of NRP1 sequence which was predicted by iTasser to be part of a short stretch of beta secondary structure. The NRP1 corresponding residues were modelled along the SEMA-4C structure with the DG residues as the anchor point. The charged residues mapped well with the corresponding residues on SNX5.

The resulting NRP1 hairpin-SNX5 complex was energy minimised and subjected to molecular dynamics simulation using GROMACS (6) (2019.2) according to the previously published procedure (7). 20 ns atomistic molecular dynamics allowed the backbone and side chain residues to relax. It was encouraging to see that the complex showed no signs of dissociating. To test this model further, residues upstream and downstream of this beta hairpin (according to the model generated with iTasser) were attached, energy-minimised and subjected to 20 ns molecular dynamics simulation as before. This extended hairpin peptide showed no signs of dissociating

under these short simulation conditions. This conformation of the NRP1-SNX5 complex became one of the models proposed for experimental exploration.

Fig. S1. Validation of the HRP-TGN46 Labelling Methodology

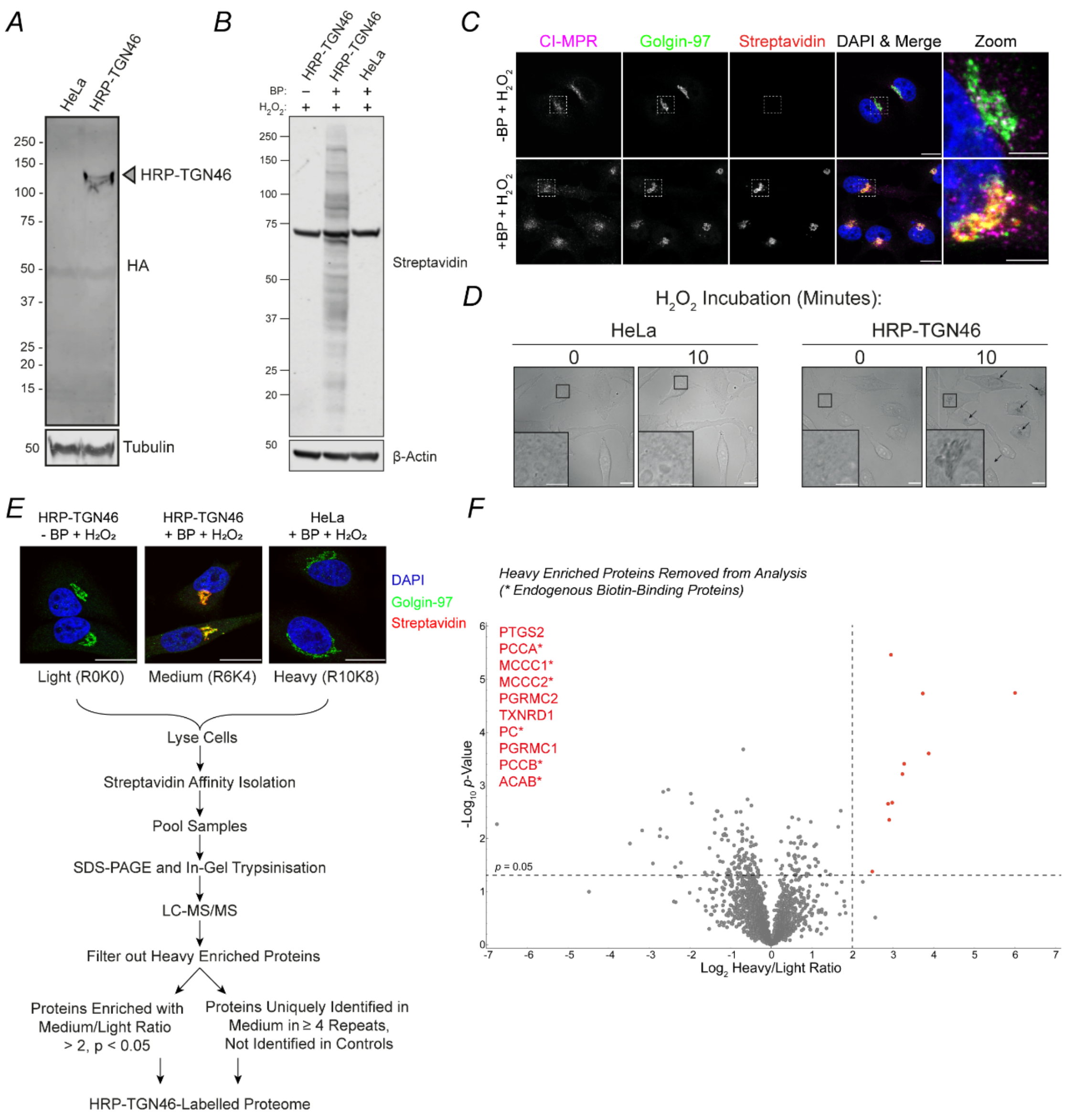

(A) Identification of HRP-TGN46 in concentrated whole cell lysates with an anti-HA antibody. **(B)** Streptavidin labelling of biotinylated proteins in HRP-TGN46-expressing HeLa cells. **(C)** Fluorescent streptavidin labelling of HRP-TGN46-expressing HeLa cells incubated with H_2O_2 in the absence or presence of biotin-phenol. The TGN marker Golgin-97 and retrograde endosomal cargo CI-MPR are labelled by immunofluorescence. Scale bars = 20 µm, 5 µm insets. **(D)** Differential interference contrast imaging of HRP-TGN46 expressing cells before and after incubation with DAB and H_2O_2. Arrows indicate electron-dense contrast after 10 minutes localising to the Golgi/TGN. Scale bar: Scale bar: 20 µm, zoom scale bar: 5 µm. **(E)** SILAC experimental design and workflow

for the identification of the HRP-TGN46-labelled proteome. Scale bars = 20 µm. **(F)** Volcano plot of proteins identified following streptavidin affinity isolation displayed as a ratio of heavy (HeLa + BP + H_2O_2) over light (HRP-TGN46 – BP + H_2O_2) abundance. Proteins significantly enriched in the heavy condition ($p < 0.05$, Log_2 fold change >2) were filtered out of subsequent analysis. Asterisks represent significantly enriched proteins with known endogenous biotin-binding affinity.

Fig. S2. Proteomic Identification of Potential ESCPE-1 Retrograde Cargoes

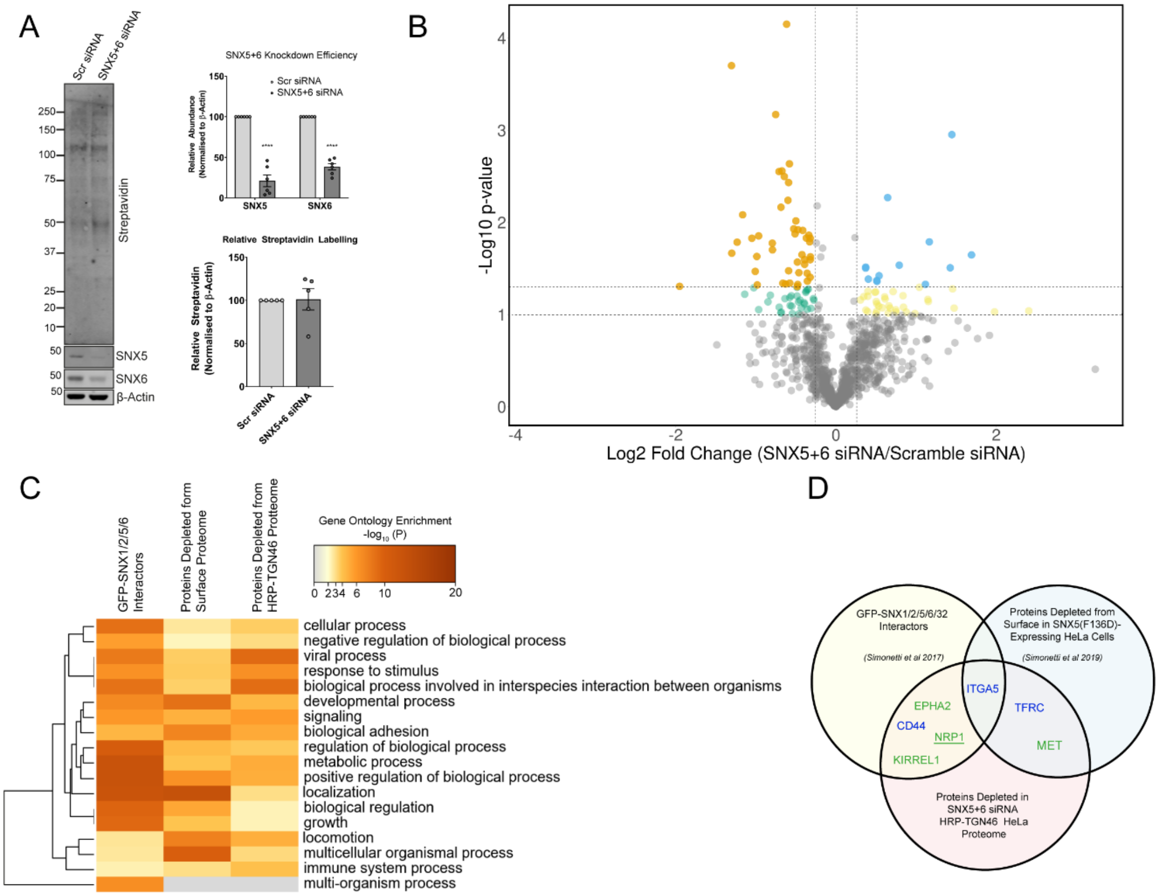

(A) Western blot of whole cell lysates from HRP-TGN46-expressing HeLa cells treated with Scr siRNA or SNX5+6 siRNA. SNX5 and SNX6 levels were quantified following normalisation to β-Actin. 2-way ANOVA with Šídák's multiple comparisons test, n=6, $p < 0.0001$ (SNX5), $p < 0.0001$ (SNX6). Total streptavidin was quantified following normalisation to β-Actin, unpaired t-test, n=5, p = 0.9272. **(B)** Volcano plot displaying HRP-TGN46 target proteins and their fold change as a ratio of SNX5+6 siRNA abundance/Scr siRNA abundance. 76 proteins are highlighted as passing a $p < 0.1$,Log_2 fold change < -0.26 threshold (green), and 46 passing a $p < 0.05$, Log_2 fold change < -0.26 threshold (orange). **(C-D)** Heat map of gene ontology term enrichment (C) and Venn diagram of overlapping proteins (D) in lists of ESCPE-1 interactors (8), cell surface cargoes (9) and proteins depleted in the HRP-TGN46 proteome ($p < 0.1$, Log_2 Fold change < -0.26) upon SNX5+6 siRNA suppression. Protein names are coloured based on the absence (blue) or presence (green) of a $\Phi x \Omega x \Phi x_n \Phi$ cytosolic sorting motif.

Fig. S3 Internalisation and Tubulovesicular Trafficking of a GFP-Nrp1 Construct

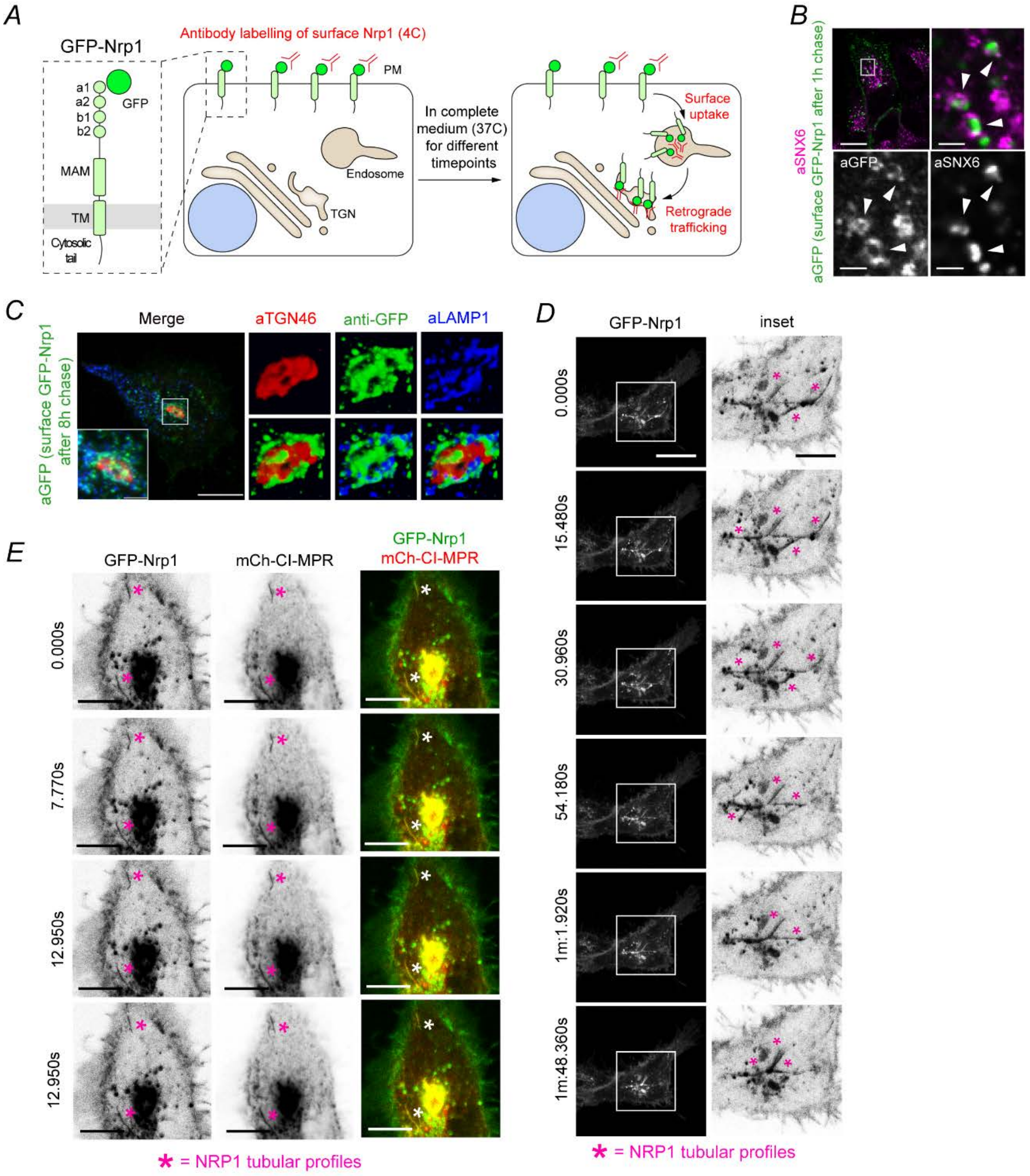

(A) Schematic of the GFP-Nrp1 uptake assay with the two-step staining procedure used to distinguish total from internalised GFP-Nrp1. **(B)** HeLa cells were transfected with GFP-Nrp1 for 24 hours and subjected to surface labelling of GFP-Nrp1. The uptake assay was followed for 1 hour. Cells were fixed and immunostained for the ESCPE-1 subunit SNX6. Arrowheads: examples of colocalisation of GFP-Nrp1 and SNX6 on tubular endosomes. Scale bar = 20 µm, Inset = 5 µm. **(C)** 8 hour surface uptake of GFP-Nrp1 in HeLa cells. The volume of the perinuclear region, including the TGN was reconstructed from serial z-stacks. Scale bar = 20 µm, Inset = 5 µm. **(D)** HeLa cells

were transfected with GFP-Nrp1 and live imaged after 24 hours. Asterisk = examples of Nrp1-positive tubular profiles that emanate from intracellular compartments. Scale bar = 20 µm, Inset = 10 µm. **(E)** HeLa cells were cotransfected with GFP-Nrp1 and mCherry-CI-MPR and live imaged after 24 hours. Asterisk = examples of Nrp1 and CI-MPR signals colocalising in tubular profiles that dynamically move within the cells. Scale bar = 10 µm.

Fig. S4 NRP1 and NRP2 Interact with ESCPE-1 Subunits

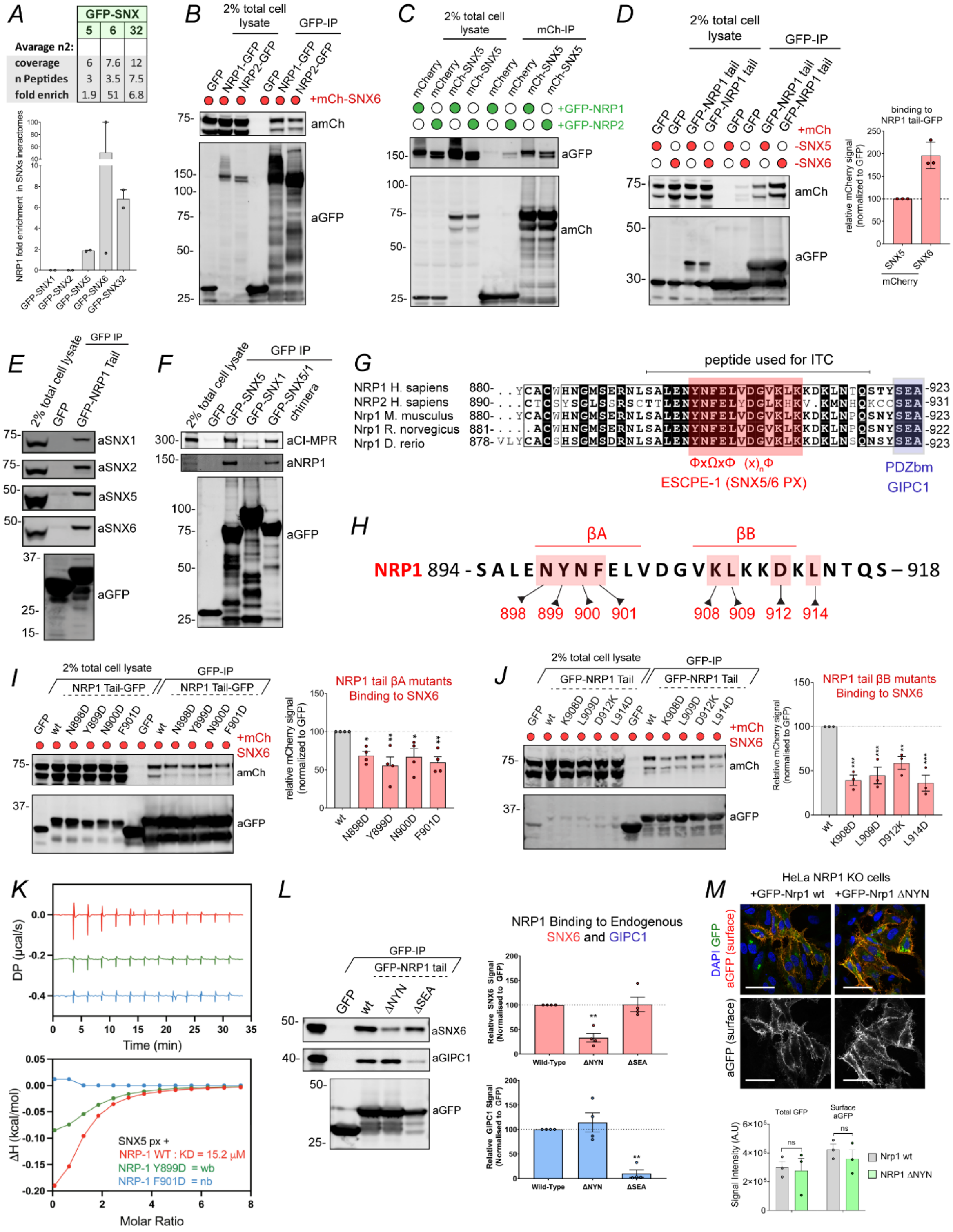

(A) Enrichment and coverage of NRP1 in the previously published SILAC-based proteomics of SNX5, SNX6 and SNX32(8). **(B)** HEK293T cells were cotransfected to express GFP-tagged NRP1 or NRP2 and mCherry-tagged SNX6 and subjected to GFP-nanotrap. The blot is representative of

three independent GFP traps. **(C)** HEK293T cells were cotransfected to express GFP-tagged NRP1 or NRP2 and mCherry or mCherry-tagged SNX5 and subjected to mCherry-nanotrap. The blot is representative of three independent mCherry traps. **(D)** HEK293T cells were cotransfected to express GFP, NRP1-GFP or NRP2-GFP, and mCherry, mCherry-SNX5 or mCherry-SNX6 and subjected to GFP-nanotrap. The band intensity of mCherry was measured from N = 4 independent experiments using Odyssey software. **(E)** HEK293T cells were transfected with GFP or GFP-NRP1 Tail, and lysates subjected to GFP nanotrap and blotting for ESCPE-1 subunits. Data representative of three independent repeats. **(F)** HEK293T cells were transfected to express GFP-tagged SNX5, SNX1 and a SNX1 chimera generated by the replacement of the SNX1 Px domain with that of SNX5. Data representative of three independent repeats. **(G)** Sequence alignment of NRP1 and NRP2 from *H. sapiens*, and Nrp1 from *M. musculus*, *R. norvegicus* and *D. rerio*. **(H)** Schematics of the NRP1 cytosolic tail sequence that accounts for the ESCPE-1 binding motif, the residues mutated in the constructs used in this study are highlighted in red. **(I)** HEK293T cells were cotransfected to express GFP-tagged wildtype or mutant forms of the NRP1 tail together with mCherry-SNX6 and subjected to GFP-nanotrap. The band intensity of mCherry was measured from N = 4 independent experiments using Odyssey software. The relative binding of mutant forms over the wild-type NRP1 tail was measured using one-way ANOVA and Dunnett's test. N898D: p = 0.0454; Y899D: p = 0.0046; N900D: p = 0.0333; F901D: p = 0.0094. **(J)** HEK293T cells were cotransfected to express GFP-tagged wildtype or mutant forms of the NRP1 tail together with mCherry-SNX6 and subjected to GFP-nanotrap. The band intensity of mCherry was measured from N = 3 independent experiments using Odyssey software. The relative binding of mutants forms the wild-type NRP1 tail was measured using one-way ANOVA and Dunnett's test. K908D: p = 0.0005; L909D: p = 0.0010; D912K: p = 0.0077; L914D: p = 0.0003. **(K)** SNX5 PX domain was titrated against different wild-type, Y899D and F901D NRP1 tail peptides and binding was measured by ITC. Top panel shows the raw data and bottom panel shows the integrated and normalised data fitted with a 1:1 binding model. The kD values were measured over N = 3. **(L)** HEK293T cells were co-transfected to express a wild-type GFP-tagged NRP1 tail construct or mutants forms lacking the ESCPE-1 binding motif (ΔNYN) or the PDZ-binding motif (ΔSEA) and subjected to GFP-nanotrap. Endogenous ESCPE-1 subunits and GIPC1 were co-immunoprecipitated and band intensities were measured from N = 4 independent experiments. One-way ANOVA with Dunnett's multiple comparisons tests. SNX6 ΔNYN p = 0.0019, SNX6 ΔSEA p = 0.9951, GIPC1 ΔNYN p = 0.6377, GIPC1 ΔSEA p = 0.0010. **(M)** Immunofluorescence staining and confocal microscopy of HeLa NRP1KO cells transfected with GFP-Nrp1 wt and GFP-Nrp1 ΔNYN. Non-permeabilised cells were labelled with anti-GFP, and signal intensity was quantified using Volocity software. Two-way ANOVA and Šídák's multiple comparisons test. Total GFP: p = 0.9550; Surface aGFP: p = 0.7244. Scale bar = 50 µm.

The bars, error bars and circles represent the mean, SEM and individual data points, respectively. $*p < 0.05$, $**p < 0.01$, $***p < 0.001$, $****p < 0.0001$.

Fig. S5 Model for ESCPE-1-Dependent Trafficking of Internalised NRP1-Bound SARS-CoV-2

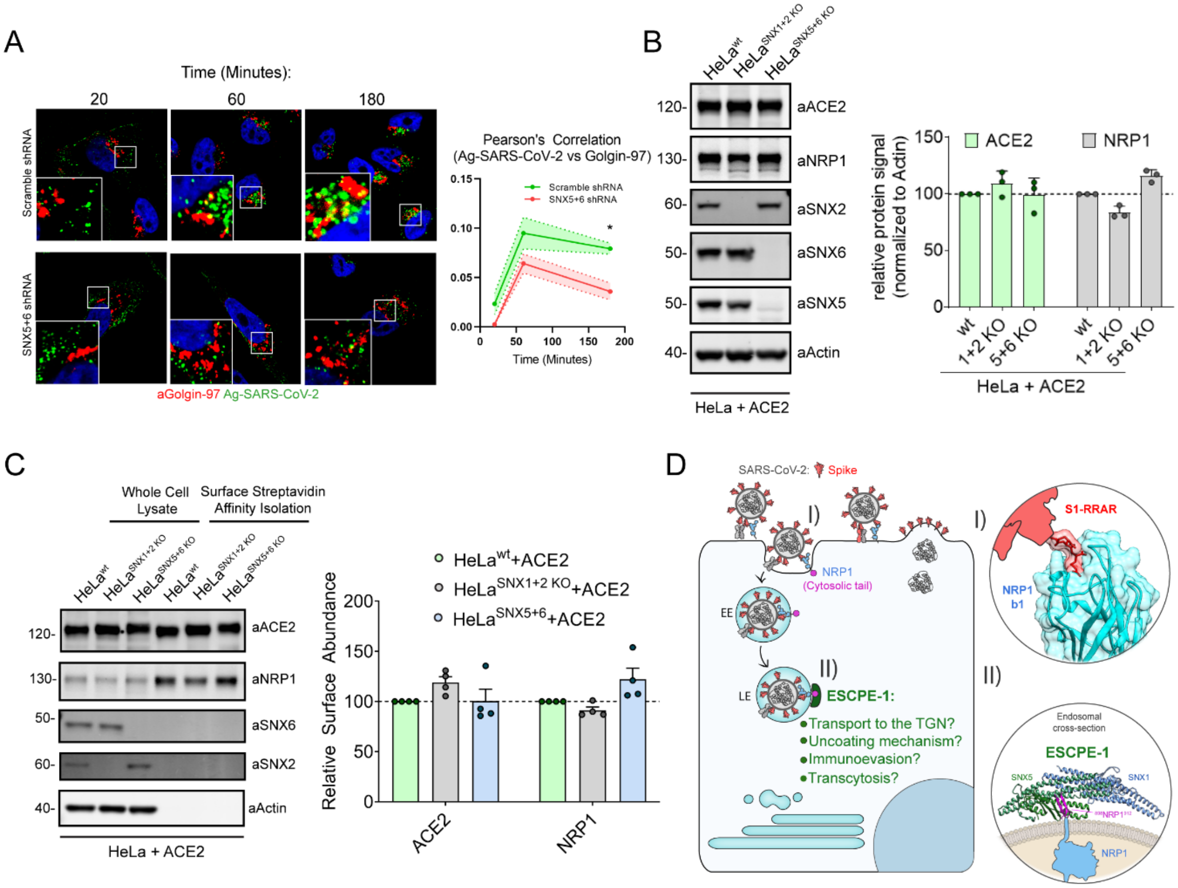

(A) Ag-SARS-CoV-2 uptake assay in Scramble shRNA PPC-1 cells or SNX5+6 shRNA PPC-1 cells. Pearson's correlation between Ag-SARS-CoV-2 and anti-Golgin-97 is measured over time using Image J software (N = 3 experiments). Two-way ANOVA with Šídák's multiple comparisons test, p = 0.4040 (20 minutes), p = 0.1316 (60 minutes), p = 0.0259 (180 minutes). Scale = 20 µm. **(B)** Quantification of ACE2 and NRP1 levels in HeLa^{wt}+ACE2, HeLa$^{SNX1+2\ KO}$+ACE2 cells and HeLa$^{SNX5+6\ KO}$ +ACE2 (n=3). ACE2 and NRP1 levels were quantified and compared to HeLa^{wt}+ACE2 using a two-way ANOVA and Dunnett's multiple comparisons tests. For ACE2 levels; HeLa^{wt}+ACE2 vs. HeLa$^{SNX1+2\ KO}$+ACE2: p = 0.3213, HeLa^{wt}+ACE2 vs. HeLa$^{SNX5+6\ KO}$: p = 0.9936. For NRP1 levels; HeLa^{wt}+ACE2 vs. HeLa$^{SNX1+2\ KO}$+ACE2: p = 0.0530, HeLa^{wt}+ACE2 vs. HeLa$^{SNX5+6\ KO}$: p = 0.0580. **(C)** Analysis of surface levels of ACE2 and NRP1 in HeLa^{wt}+ACE2, HeLa$^{SNX1+2KO}$+ACE2 cells and HeLa$^{SNX5+6KO}$+ACE2 cells. Cells were subjected to surface biotinylation followed by streptavidin-based affinity isolation and blotting for ACE2 and NRP1 (n=4). Receptor levels were compared to HeLa^{wt}+ACE2 using a two-way ANOVA with Dunnett's multiple comparisons tests. For ACE2 levels; HeLa^{wt}+ACE2 vs. HeLa$^{SNX1+2\ KO}$+ACE2: p = 0.1346, HeLa^{wt}+ACE2 vs. HeLa$^{SNX5+6\ KO}$: p = 0.9981. For NRP1 levels; HeLa^{wt}+ACE2 vs. HeLa$^{SNX1+2\ KO}$+ACE2: p = 0.5970, HeLa^{wt}+ACE2 vs. HeLa$^{SNX5+6\ KO}$: p = 0.0744. **(D)** Model for ESCPE-1-

dependent trafficking of Internalised SARS-CoV-2. (I) SARS-CoV-2 engages NRP1 at the cell surface via direct binding between the CendR motif of S1 and the extracellular NRP1 b1 domain. (II) Once internalised into the endosomal network, the cytosolic tail of NRP1 is directly recognised by ESCPE-1, facilitating ESCPE-1 coat oligomerisation, membrane deformation and coupling to the cytoskeleton, along with potential consequences for SARS-CoV-2 biology.

Movie S1 (separate file).

HeLa cells were transfected with GFP-Nrp1. 24 hours after transfection, cells were live imaged using a confocal laser-scanning microscope at 37ºC and incidences of GFP-Nrp1 (greyscale) localisation on tubular structures were observed. Representative frames are displayed in Fig. S3D.

Movie S2 (separate file).

HeLa cells were co-transfected with GFP-Nrp1 and mCherry-SNX1. 24 hours after transfection, cells were live imaged using a confocal laser-scanning microscope at 37ºC and incidences of GFP-Nrp1 (green) and mCherry-SNX1 (red) colocalisation on tubular structures were observed. Representative frames are displayed in Figure 3D.

Movie S3 (separate file).

HeLa cells were co-transfected with GFP-Nrp1 and mCherry-SNX1. 24 hours after transfection, cells were live imaged using a confocal laser-scanning microscope at 37ºC and incidences of GFP-Nrp1 (green) and mCherry-SNX1 (red) colocalisation on tubular structures were observed. Representative frames are displayed in Fig. 3E.

Dataset S1 (separate file).

Complete list of proteins identified by SILAC-based proteomics following proximity labelling by HRP-TGN46. Untransfected HeLa cells were labelled in light (R0K0) SILAC media and treated with BP + H_2O_2, HRP-TGN46-expressing HeLa cells were labelled in medium (R6K4) SILAC media and treated with BP + H_2O_2, and HRP-TGN46-expressing HeLa cells were treated with BP in the absence of H_2O_2. N = 5 independent replicates. 10 proteins that were significantly enriched in the heavy (HRP-TGN46 expressing cells incubated with BP in the absence of H_2O_2) relative to untransfected HeLa cells, some of which were endogenous biotin- binding proteins, are highlighted in red and were removed from subsequent analyses.

Dataset S2 (separate file).

Filtered list of proteins reproducibly enriched by HRP-TGN46 biotinylation. Proteins that were statistically significantly enriched in HRP-TGN46 expressing cells (medium condition) relative to untransfected HeLa cells (light condition) ($p < 0.05$, Log_2 fold change >2), or present in ≥ 4 medium samples but < 3 light samples are presented.

Dataset S3 (separate file).

Gene ontology analysis of enriched cellular components in the HRP-TGN46-labelled proteome. The list of proteins labelled by HRP-TGN46 **(Dataset S2)** was analysed with PANTHER gene ontology software. The top 50 significantly enriched/depleted cellular component categories are displayed, ranked by p-value. + = significant overrepresentation, - = significant underrepresentation of categories.

Dataset S4 (separate file).

Gene ontology analysis of enriched biological processes in the HRP-TGN46-labelled proteome. The list of proteins labelled by HRP-TGN46 **(Dataset S2)** was analysed with PANTHER gene ontology software. The top 50 significantly enriched/depleted biological process categories are displayed, ranked by p-value. + = significant overrepresentation, - = significant underrepresentation of categories.

Dataset S5 (separate file).

Enrichment of mannose-6-phosphate (M6P)-tagged proteins in the HRP-TGN46-labelled proteome. M6P-tagged proteins identified in (10) that were significantly enriched compared to untransfected HeLa cells (light condition) are displayed.

Dataset S6 (separate file).

Complete list of proteins identified by SILAC-based proteomics following proximity labelling by HRP-TGN46 in Scr and SNX5+SNX6 siRNA-treated cells. Scramble siRNA-treated cells were labelled in light (R0K0) SILAC media, and SNX5+6 siRNA-treated cells were labelled in medium (R6K4) SILAC media, both conditions were biotinylated with BP and H_2O_2 and subjected to streptavidin affinity isolation. N = 4 independent replicates.

Dataset S7 (separate file).

Filtered list of identified proteins from siRNA suppression screen. The list of proteins identified by SILAC-based proteomics in Dataset S6 was filtered to only include proteins present in the previously established HRP-TGN46-labelled proteome in Dataset S2.

Dataset S8 (separate file).

List of enriched and depleted proteins in the SNX5+6 siRNA HRP-TGN46-Labelled proteome. Proteins with a fold change of $Log_2 \pm 0.26$ and a p-value of < 0.1 are displayed. Proteins that were depleted from the SNX5+6 siRNA proteome relative to the Scr siRNA proteome are classified by the presence of a transmembrane domain, and putative cytosolic ΩxΦxΩ SNX5/6 interacting motifs. Proteins that were either identified as ESCPE-1 interactors (8) or depleted from a GFP-SNX5(F136D) surface proteome (9) are also indicated.

Dataset S9 (separate file).

Gene ontology analysis of cellular component categories depleted in the SNX5+6 siRNA HRP-TGN46-labelled proteome. The list of proteins depleted in the SNX5+6 siRNA HRP-TGN46 proteome (**Dataset S8**) were analysed with PANTHER gene ontology software. Significantly enriched/depleted biological process categories are displayed, ranked by p-value. + = significant overrepresentation, - = significant underrepresentation of categories.

Dataset S10 (separate file).

Gene ontology analysis of biological process categories depleted in the SNX5+6 siRNA HRP-TGN46-labelled proteome. The list of proteins depleted in the SNX5+6 siRNA HRP-TGN46 proteome (**Dataset S8**) were analysed with PANTHER gene ontology software. Significantly enriched/depleted biological process categories are displayed, ranked by p-value. + = significant overrepresentation, - = significant underrepresentation of categories.

Dataset S11 (separate file).

Gene ontology analysis of molecular function categories depleted in the SNX5+6 siRNA HRP-TGN46-labelled proteome. The list of proteins depleted in the SNX5+6 siRNA HRP-TGN46 proteome **(Dataset S8)** were analysed with PANTHER gene ontology software. Significantly

enriched/depleted biological process categories are displayed, ranked by p-value. + = significant overrepresentation, - = significant underrepresentation of categories.

SI References

1. L. Zimmermann, *et al.*, A Completely Reimplemented MPI Bioinformatics Toolkit with a New HHpred Server at its Core. *J. Mol. Biol.* **430**, 2237–2243 (2018).
2. H. M. Berman, *et al.*, The Protein Data Bank. *Nucleic Acids Res.* **28**, 235–242 (2000).
3. N. Gibbs, A. R. Clarke, R. B. Sessions, Ab Initio Protein Structure Prediction Using Physicochemical Potentials and a Simplified Off-Lattice Model (2001) https:/doi.org/10.1002/1097-0134 (December 15, 2021).
4. A. Roy, A. Kucukural, Y. Zhang, I-TASSER: a unified platform for automated protein structure and function prediction. *Nat. Protoc. 2010 54* **5**, 725–738 (2010).
5. F. Madeira, *et al.*, The EMBL-EBI search and sequence analysis tools APIs in 2019. *Nucleic Acids Res.* **47**, W636–W641 (2019).
6. D. Van Der Spoel, *et al.*, GROMACS: Fast, flexible, and free. *J. Comput. Chem.* **26**, 1701–1718 (2005).
7. C. Toelzer, *et al.*, Free fatty acid binding pocket in the locked structure of SARS-CoV-2 spike protein. *Science (80-.).* **370**, 725–730 (2020).
8. B. Simonetti, C. M. Danson, K. J. Heesom, P. J. Cullen, Sequence-dependent cargo recognition by SNX-BARs mediates retromer-independent transport of CI-MPR. *J. Cell Biol.* **216**, 3695–3712 (2017).
9. B. Simonetti, *et al.*, Molecular identification of a BAR domain-containing coat complex for endosomal recycling of transmembrane proteins. *Nat. Cell Biol.* **21**, 1219–1233 (2019).
10. T. Čaval, *et al.*, Targeted Analysis of Lysosomal Directed Proteins and Their Sites of Mannose-6-phosphate Modification. *Mol. Cell. Proteomics* **18**, 16–27 (2019).
